# The single-cell transcriptome of mTECs and CD4^+^ thymocytes under adhesion revealed heterogeneity of mTECs and a network controlled by *Aire* and lncRNAs

**DOI:** 10.3389/fimmu.2024.1376655

**Published:** 2024-08-26

**Authors:** Cíntia J. Monteiro, Max J. Duarte, Mayara Cristina V. Machado, Romário S. Mascarenhas, Patrícia V. Bonini Palma, Henry D. Mogollón García, Helder I. Nakaya, Thiago M. Cunha, Eduardo A. Donadi, Geraldo A. Passos

**Affiliations:** ^1^ Molecular Immunogenetics Group, Department of Genetics, Ribeirão Preto Medical School, University of São Paulo (USP), Ribeirão Preto, SP, Brazil; ^2^ Blood Center of Ribeirão Preto, Ribeirão Preto Medical School, University of São Paulo (USP), Ribeirão Preto, SP, Brazil; ^3^ Institute of Biology, Campinas State University (UNICAMP), Campinas, SP, Brazil; ^4^ Research Institute, Albert Einstein Israeli Hospital, São Paulo, SP, Brazil; ^5^ Department of Clinical and Toxicological Analysis, School of Pharmaceutical Sciences, University of São Paulo, São Paulo, Brazil; ^6^ Center for Research in Inflammatory Diseases, Department of Pharmacology, Ribeirão Preto Medical School, University of São Paulo (USP), Ribeirão Preto, SP, Brazil; ^7^ Department of Medicine, Division of Clinical Immunology, Ribeirão Preto Medical School, University of São Paulo (USP), Ribeirão Preto, SP, Brazil; ^8^ Department of Basic and Oral Biology, Ribeirão Preto School of Dentistry, University of São Paulo (USP), Ribeirão Preto, SP, Brazil; ^9^ Center for Cell-Based Therapy in Dentistry, Ribeirão Preto School of Dentistry, University of São Paulo (USP), Ribeirão Preto, SP, Brazil

**Keywords:** *Aire*, medullary thymic epithelial cells, cell adhesion, single-cell transcriptome, long noncoding RNAs

## Abstract

To further understand the impact of deficiency of the autoimmune regulator (*Aire*) gene during the adhesion of medullary thymic epithelial cells (mTECs) to thymocytes, we sequenced single-cell libraries (scRNA-seq) obtained from *Aire* wild-type (WT) (*Aire^wt/wt^*) or *Aire*-deficient (*Aire^wt/mut^*) mTECs cocultured with WT single-positive (SP) CD4^+^ thymocytes. Although the libraries differed in their mRNA and long noncoding RNA (lncRNA) profiles, indicating that mTECs were heterogeneous in terms of their transcriptome, UMAP clustering revealed that both mTEC lines expressed their specific markers, i.e., *Epcam, Itgb4*, *Itga6*, and *Casp3* in resting mTECs and *Ccna2, Pbk*, and *Birc5* in proliferative mTECs. Both cocultured SP CD4^+^ thymocytes remained in a homogeneous cluster expressing the *Il7r* and *Ccr7* markers. Comparisons of the two types of cocultures revealed the differential expression of mRNAs that encode transcription factors (*Zfpm2, Satb1*, and *Lef1*), cell adhesion genes (*Itgb1*) in mTECs, and Themis in thymocytes, which is associated with the regulation of positive and negative selection. At the single-cell sequencing resolution, we observed that *Aire* acts on both *Aire* WT and *Aire*-deficient mTECs as an upstream controller of mRNAs, which encode transcription factors or adhesion proteins that, in turn, are posttranscriptionally controlled by lncRNAs, for example, Neat1, Malat1, Pvt1, and Dancr among others. Under *Aire* deficiency, mTECs dysregulate the expression of MHC-II, CD80, and CD326 (EPCAM) protein markers as well as metabolism and cell cycle-related mRNAs, which delay the cell cycle progression. Moreover, when adhered to mTECs, WT SP CD4^+ ^or CD8^+^ thymocytes modulate the expression of cell activation proteins, including CD28 and CD152/CTLA4, and the expression of cellular metabolism mRNAs. These findings indicate a complex mechanism through which an imbalance in *Aire* expression can affect mTECs and thymocytes during adhesion.

## Graphical Abstract

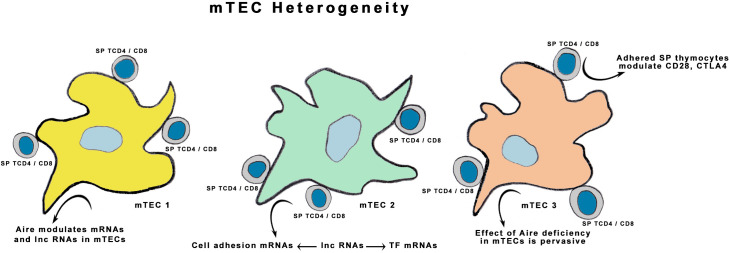

## Introduction

Adhesion between medullary thymic epithelial cells (mTECs) and thymocytes is a crucial step for single-positive (SP) CD4^+^ or CD8^+^ thymocytes recognize self-antigen peptides presented by mTECs through MHC-II molecules. In parallel, thymocyte clones that express TCRα/β with high affinity/avidity for self-antigens are eliminated by apoptosis (1–5). These processes are known as thymic crosstalk and negative selection of autoreactive thymocytes, which represent the basis of central immune tolerance induction (6–8).

mTECs are peculiar cells, as they express approximately 80% of their functional genome in terms of mRNAs, maintaining their morpho functional identity, which is important for thymocyte attraction and adhesion (9–11). The autoimmune regulator (*Aire*) gene encodes the AIRE protein, a transcriptional autoantigen controller that primarily controls mTEC transcription. Within the thymus, it is exclusively expressed in the medulla by mTECs (12–14). A second transcriptional regulator in mTECs that functions as a classical transcription factor is the FEZ family zinc finger 2 (*Fezf2*) gene (15, 16). Although the AIRE and FEZF2 proteins act differently on chromatin, their final effects seem to be synergistic since they control transcription in mTECs.

AIRE pushes RNA polymerase II (RNA Pol II) anchored to the chromatin of mTECs, continuing the transcription elongation phase (17). This process permits RNA Pol II to transcribe mRNAs, miRNAs, and long noncoding RNAs (lncRNAs) (18, 19). The lncRNAs were chosen for the study due to previous observations from our group showing that *Aire* controls this RNA species in mTECs (18). The lncRNAs are transcribed by the RNA Pol II, but unlike mRNAs, they do not serve as templates for protein synthesis. Their function is associated with the control of gene expression at different levels, such as its association with chromatin for transcription controlling, lncRNA-mRNA interaction for mRNA translation controlling, and the lncRNA-protein interaction. Therefore, lncRNAs can interact with all types of informational molecules in mammalian cells whose function is associated with the control of gene expression flow, chromatin remodeling, and epigenetic regulation (20, 21). In turn, the lncRNAs are capable of mutual interactions and could result in posttranscriptional control of mTEC gene expression.

These processes result in the thymic medullary expression of virtually all self-antigens, representing all tissue and organ antigens; this process is termed promiscuous gene expression (PGE) (11, 13, 14, 22, 23). However, PGE in mTECs is stochastic; each cell expresses a small group of autoantigens that characterize a given organ (23–25). Therefore, self-representation in the thymus must be interpreted as a whole mTEC population process; i.e., the body’s self-representation is made by the whole set of mTECs, which guarantees immunological tolerance (26, 27).

Our group has been working on the influence of *Aire* or noncoding RNAs on cell adhesion-related mRNAs and the mTEC–thymocyte adhesion process (18, 28, 29). We showed that *Aire* controls the expression of proteins involved in cell-cell adhesion and that when *Aire* is deficient, mTECs disturb their adhesion to thymocytes. We also observed that *Aire* controls i) the expression of miRNAs in mTECs (30–32), ii) the expression of lncRNAs modulated by *Aire* when mTECs adhere to thymocytes (18), iii) the expression of specific miRNAs controlling mTEC–thymocyte adhesion (28), and iv) the ability of miR-155 to control *Aire* (33).

In this study, we hypothesize that *Aire* deficiency in mTECs is pervasive. In addition to affecting the mTECs’ transcriptome, which affects their heterogeneity, the thymocytes that adhere to these cells could also present disturbances in their transcriptional expression.

Using single-cell RNA-seq, a suitable method for dissecting cell heterogeneity (34), we have uncovered a novel aspect of *Aire* deficiency. Results show that *Aire* deficiency not only modifies the mTEC clusters but also triggers a unique transcriptional response in SP CD4^+^ thymocytes upon their interaction with these cells. This response is mediated by intricate interactions between lncRNAs and mRNAs encoding transcription factors or adhesion molecules.

## Materials and methods

### Mice

The wild-type (WT) C57BL/6J *Mus musculus* mice were obtained from Jackson Laboratory (https://www.jax.org/) and bred and maintained at the Central Animal Facility, University of São Paulo, Ribeirão Preto Campus, SP, Brazil, under specific-pathogen-free conditions in 0.45-μm air-filtered ventilated racks at a constant temperature of approximately 22°C under 12-h dark/light cycles and receiving water and food *ad libitum*. The animals were killed by CO_2_ aspiration in an acrylic chamber. The thymus gland was removed by thoracic surgery, and single-positive (SP) CD4^+^ or CD8^+^ thymocytes were separated through flow cytometry cell sorting using specific antibodies as described below. This work was approved by the Ethics Committee for Animal Research, Ribeirão Preto Medical School, University of São Paulo (CEUA approval # 003/2017-1).

### Flow cytometry

For flow cytometry analysis and sorting, we used a BD FACS Calibur apparatus (Beckton Dickinson Biosciences, Franklin Lakes, NJ) and the following antibodies: APC-Cy7 rat anti-mouse CD8a (Cat # 557654, BD Pharmingen, Franklin Lakes, NJ), PE-labeled rat IgG2b, k anti-mouse CD4 antibody (BioLegend, San Diego, CA, Cat # 100512), APC rat anti-mouse CD326 (Epcam) (Cat # 118214, BioLegend), FITC-labeled rat anti-mouse I-A/I-E (MHC-II) (Cat # 553623, Becton Dickinson), and PE-Cy7anti-mouse CD80 monoclonal antibody (Cat # 12-0801-82, Invitrogen, Waltham, MA). For data analysis, we used the bundled BD FACSDiva software (https://www.bdbiosciences.com/en-us/products/software/instrument-software/bd-facsdiva-software).

### Cells and cocultures

We employed the murine (*M. musculus*) *Aire* WT mTEC 3.10 line (EpCAM^+^, Ly51^-^, UEA1^+^), as previously described (35, 36). These cells express the *Aire*, *Ccl21*, and *Sap1* mRNAs (19) and the AIRE protein (18, 33). Moreover, we previously showed via immunofluorescence that the WT mTEC 3.10 line expresses *Aire* mRNA and the AIRE protein, which is localized in the cell nucleus (37, 38). According to an mTEC phenotypic classification proposed (39), the mTEC 3.10 cell line is compatible with the mTEC^low^ differentiation stage.

In addition, we used a CRISPR-Cas9-generated heterozygous *Aire* mutant (Aire^wt/mut^), here termed *Aire*-deficient, which was derived from the WT mTEC 3.10 cell line and is known as the mTEC CS8D6 clone (40) This clone is a carrier of an indel NHEJ-derived mutation (del 3554G) in the *Aire* exon 6, which encodes the SAND domain of the AIRE protein. The FASTA sequence of part of the *Aire* exon 6 gene, which encodes the SAND domain harboring the del 3554G mutation, is available at the GenBank NCBI (https://www.ncbi.nlm.nih.gov/nucleotide/) under accession number PP034558.

For mTEC–thymocyte cocultures, we followed a protocol as previously described (18) with modifications. In brief, the mTECs were cultured in RPMI 1640 medium containing 10% inactivated fetal bovine serum and antibiotics at 37°C in a 5% CO_2_ atmosphere. Semiconfluent cultures were detached from their culture flasks by conventional trypsin/EDTA treatment, washed once with sterile PBS at room temperature, resuspended in RPMI 1640 medium, and seeded in new culture flasks (2 × 10^6^ cells per 75 cm^2^ Corning^®^ cell culture flasks).

For this study, *Aire* WT or *Aire*-deficient mTECs were cocultured with thymocytes, as follows: The WT SP CD4^+^ or CD8^+^ thymocytes were added to mTECs at a ratio of 5:1 (thymocyte:mTEC) and cocultured in RPMI medium containing 10% inactivated fetal bovine serum and antibiotics at 37°C in a 5% CO_2_ atmosphere for 36 hours. Next, the nonadherent thymocytes were carefully removed from cultures by washing with PBS at 37°C and discarded. The culture flasks were washed more vigorously with PBS at 4°C to remove the adherent thymocytes, which were kept for counting.

The mTEC cells were detached from their culture flasks by conventional trypsin/EDTA treatment and resuspended in PBS for counting. Cell counts for either thymocytes or mTECs were performed on a Cellometer Auto T4 Cell Viability Counter (Nexcelon Bioscience, Lawrence, MA, USA). However, estimating the individual adhesion strength of each mTEC-thymocyte pair is impossible, as is determining whether this effected the transcriptional response of these cells. However, our methodology ensures that CD4+ T cells not adhering to mTECs were not collected since they were removed through washing with PBS, thereby focusing our analysis on the relevant interacting cells. While there may be differences in binding affinities, these variations were consistent across all samples. Thus, maintaining uniform experimental conditions mitigated any impact on our study from differing affinities.

After incubation, the cocultures with SP CD4^+^ thymocytes were subjected to single-cell library preparation as described below. Only cocultures with SP CD4^+^ thymocytes were used to prepare single-cell libraries. The cocultures with SP CD8^+^ thymocytes were used for cell surface markers quantifying mTECs.

### Western blotting for the AIRE protein

Western blotting of the AIRE protein was performed according to a protocol adjusted in our laboratory as previously described (38), except that in this study, we changed the anti-AIRE primary and secondary antibodies.

The *Aire* WT or *Aire*-deficient mTECs were lysed using RIPA lysis and extraction buffer (Cat # 89900, Thermo-Fisher) supplemented with a complete protease inhibitor cocktail (Cat # 539134, Calbiochem, Billerica, MA). The supernatant from a 10,000x g centrifugation at 4°C for 10 minutes was collected, and cell lysates were heated at 95°C for 5 minutes in 2 x SDS-Laemmli buffer (Cat # 161073, Bio-Rad). Aliquots containing 50 μg total protein were resolved on 10% SDS-PAGE, and bands were electro-transferred onto a PVDF membrane (Cat # 162-0177, Immun-Blot PVDF membrane, Bio-Rad) using a Bio-Rad Mini Trans-Blot transfer system, following the manufacturer’s protocol. The transferred membranes were blocked with 5% low-fat milk in PBS with 0.1% Tween 20 (TBS-T) for 1 hour, washed twice in PBS, and then incubated overnight at 4°C with rabbit anti-mouse AIRE polyclonal primary antibody (Cat # orb228738, Biorbyt, Durham, NC). After washing with TBS-T, the blots were incubated for 1 hour with goat anti-rabbit HRP-conjugated secondary IgG (Cat # 31460, Thermo-Fisher). Immunoreactive bands were visualized using a chemiluminescent substrate (Cat # WBLUF0500, Immobilon^®^ Forte, Millipore), and protein bands were detected using the ImageQuant™ LAS 500 system (GE Life Sciences, Piscataway, NJ, USA). After washing to remove the primary and secondary antibodies, the membrane was incubated with an anti-GAPDH rabbit primary antibody (Cat # 2118, Cell Signaling Technology, Beverly, MA), following incubation with a peroxidase-conjugated anti-rabbit antibody. The membrane was developed as described above.

### Cell cycle analysis

The cell cycle progression of mTECs was evaluated by using the conventional propidium iodide protocol (Cat # BMS 500PI, Thermo Fisher, Waltham, MA) and analyzed in a FACS Calibur apparatus in the PE channel as previously described (29). Briefly, the samples were resuspended in 200 μL of the propidium iodide (5 μg/mL) solution and maintained for 30 min at 37°C. At least three independent experiments were analyzed.

### Single-cell library preparation and RNA sequencing

The preparation of single-cell libraries was performed according to the 10X Genomics (https://www.10xgenomics.com/) protocol (https://assets.ctfassets.net/an68im79xiti/1eX2FPdpeCgnCJtw4fj9Hx/7cb84edaa9eca04b607f9193162994de/CG000204_ChromiumNextGEMSingleCell3_v3.1_Rev_D.pdf) with Chromium Next GEM single cell 3´ technology, paired, library and gel bead kit v3.1 (PN-1000128) and a Chromium instrument (10X Genomics, Pleasanton, CA). In brief, the single cells were isolated and lysed, and the mRNAs were purified and primed with a poly(T) primer for reverse transcription. Unreactive primers were removed by exonuclease I digestion. Poly(A) tails were added to the first-strand cDNA at the 3’ end and annealed to poly(T) primers for second-strand cDNA generation. The cDNAs were PCR-amplified, sheared, and prepared into a sequencing library.

The library from the coculture of *Aire*-deficient mTECs with WT SP CD4^+^ thymocytes yielded 8,391 cells and a mean of 30,641 reads per cell. The library from the coculture of *Aire* WT mTECs with WT SP CD4^+^ thymocytes yielded 1,991 cells and a mean of 106,003 reads per cell. For the comparisons between the two libraries, the duplets were identified and removed, and the values were normalized using the SCTransform function within the Seurat package (https://satijalab.org/seurat/).

Libraries were prepared from cocultures of *Aire*-deficient cells (*Aire* ^wt/mut^, clone CS8D6) (40) with WT SP CD4^+^ thymocytes (one library) or *Aire* WT (*Aire* ^wt/wt^) mTEC 3.10 cells with WT SP CD4^+^ thymocytes (one library). The SP CD4^+^ thymocytes were separated by flow cytometry from the thymus of one four-week-old female WT C57BL/6J mouse using a BD FACS Calibur and a primary FITC-conjugated rat anti-mouse CD4 antibody (Cat # 269349; Abcam, Waltham, MA) according to a previously described protocol (41, 42). The viability of the cocultured cells was checked with an automated Cellometer Auto T4 Cell Counter (Viability Counter Nexcelom Bioscience, Lawrence, MA), whose cocultures presented 94.2% viability (*Aire* WT mTEC 3.10 cells cocultured with WT SP CD4^+^ thymocytes) and 98.4% viability (*Aire*-deficient mTECs cocultured with WT SP CD4^+^ thymocytes) just before being used to prepare the libraries.

Total RNA samples were prepared using the mirVana kit (Ambion, Grand Island, NY) following the manufacturer’s instructions. The integrity and quality of the RNAs and the respective cDNA libraries were assessed using microfluidic electrophoresis on an Agilent Bioanalyzer (Model 2100) with an RNA nano chip 6000 or a DNA LabChip (Agilent, Santa Clara, CA). Only phenol- and protein-free samples with an RNA integrity number (RIN) ≥ 9.0 were used. The RNAs of the two libraries were sequenced on a flow-cell SP 150PE NovaSeq 6000 platform (Illumina, San Diego, CA) (paired and coverage of approximately 200 million reads per sample). The raw sequence reads are available at ncbi.nlm.nih.gov (BioProject PRJNA1001046).

### Bioinformatics analysis of single-cell RNA-seq data

The demultiplexing of the raw data reads was performed using the demultiplexing program (https://demultiplex.readthedocs.io/en/latest/). For the analysis and exploration of the scRNA-seq data, the Seurat v2.3.4 package was used (https://satijalab.org/seurat/articles/install.html) (43–45) in the R environment (https://www.r-project.org/). We used the Seurat FindAllMarkers function (https://satijalab.org/seurat/) to identify the DE mRNAs between clusters (log fold change threshold = 0.25). This package carries out quality control of cells and mRNAs, identification of the most variable mRNAs, and cell clustering. We use the Seurat package that contains the SCTransform tool for normalization based on negative binomial regression models and calculation of Pearson residuals, which takes into account scale factors (number of cells/depth) in addition to other variables techniques such as, for example, the percentage of mitochondria (percent.mt) and the difference between the observed vs expected counts by the adjusted model. Moreover, the datasets were normalized independently, and an adjustment was made to the reading depth separately, allowing the data to be compared even though they were of different sizes. The selection of marker mRNAs and cell type identification were based on the literature (44, 45). With respect to quality, only mRNAs expressed in at least three cells and cells with at least 200 expressed mRNAs and the presence of up to 10% mitochondrial DNA were considered for analysis. Clustering was performed considering 30 PCs (Seurat standard) with a resolution of 0.1.

Functional enrichment analysis of the mRNA transcripts was carried out with the Gene Ontology Panther tool (https://www.pantherdb.org/). The differentially expressed (DE) lncRNAs identified in this study that were already enriched and validated by other studies were identified via the LncTAR 2.0 platform (https://lnctard.bio-database.com/). The posttranscriptional interaction networks between lncRNAs, adhesion molecules, and transcription factor mRNAs associated with the respective biological functions were drawn using the Cytoscape version 3.10.1 tool (https://cytoscape.org/). The input-validated lncRNAs and mRNAs data were merged to reconstruct the interaction networks. The resulting networks allow one to identify the interactions between these types of RNAs, their biological functions, and the cells involved.

## Results

### Single-cell RNA-seq analysis of Aire wild-type mTEC 3.10 cells and wild-type single-positive CD4^+^ thymocytes under adhesion conditions

**Figure 1:**
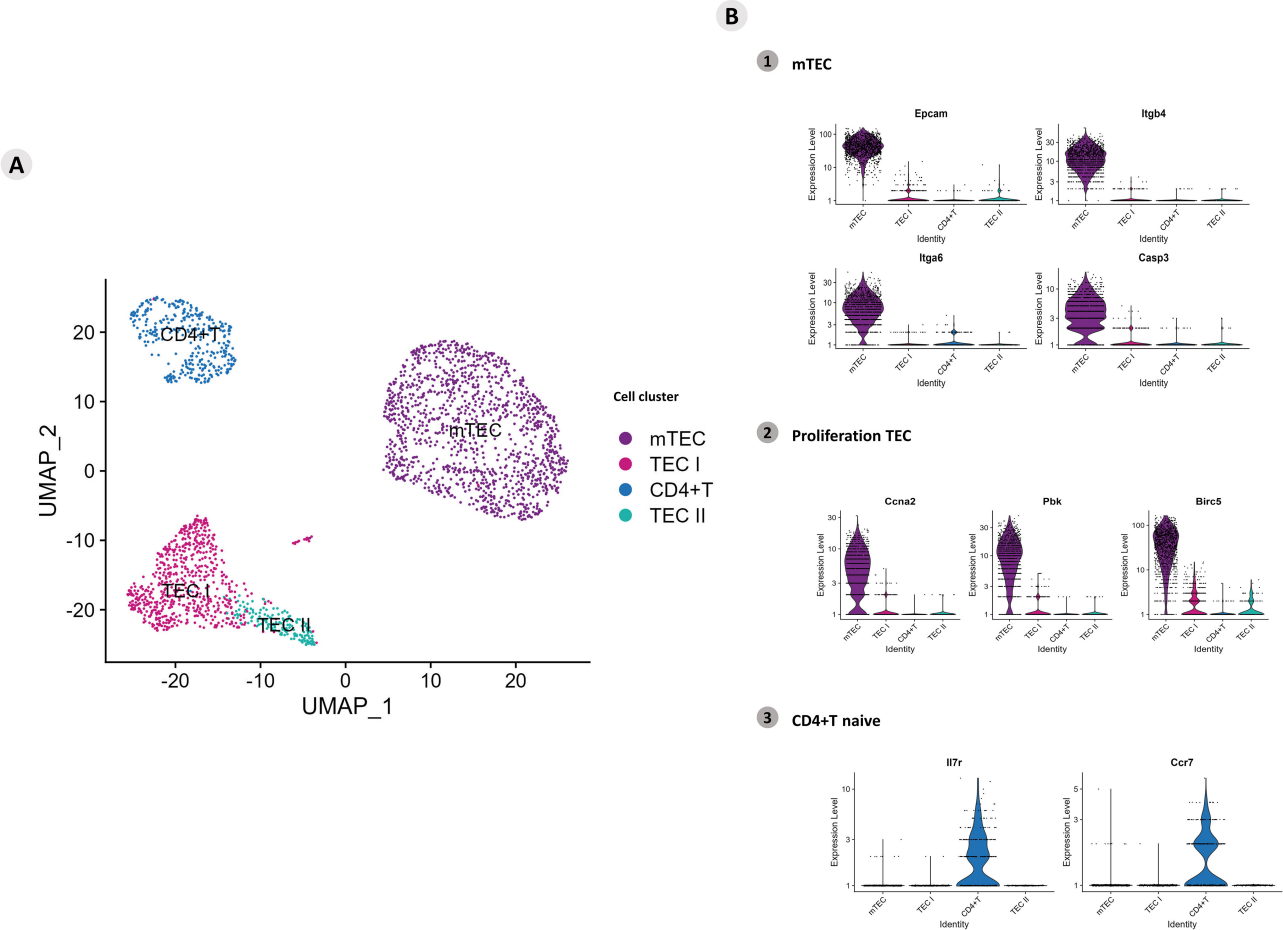
Single-cell mRNA transcriptional analysis of *Aire* wild-type medullary thymic epithelial cells cocultured with wild-type naïve CD4^+^ thymocytes. **(A)** UMAP clustering of TECs (TEC I, TEC II, mTECs, and wild-type CD4^+^ thymocytes) based on their transcriptional profiles. **(B)** mTECs were identified based on their expression of *Epcam*, *Itgb4*, *Itgb6* and *Casp3* mRNA markers (B1), proliferative TECs based on *Ccna2*, *Pbk* and *Birc5* expression (B2), and naïve CD4^+^ thymocytes based on *Ilr7* and *Ccr7* expression (B3).

The UMAPs allowed us to investigate the clusters of *Aire* wild-type (WT) mTEC 3.10 cells and WT single-positive (SP) CD4^+^ thymocytes under coculture. Four groups of cells were identified according to the expression of specific transcriptional markers on TECs (mTECs, TEC I, and TEC II) or CD4^+^ T cells (**Figure 1A**). To identify mTECs, the expression of the classic marker mRNAs *Epcam*, *Itgb4*, *Itgb6*, and *Casp3* (*Sca1*) was evaluated (**Figure 1**; **Supplementary Figure 1**). The marker mRNAs *Ccna2*, *Pbk*, and *Birc5* characterize proliferative TECs, in addition to two other clusters that characterize the TEC I and TEC II groups (**Supplementary Figure 2**). Naïve CD4^+^ T cells were identified; these cells remained in a cluster expressing the respective mRNAs that encode *Il7r* or *Ccr7* (**Figure 1**). The mRNAs with high DE values are highlighted and shown in the heatmap (top mRNAs). The transcription of mRNAs with more significant variability is highlighted in the heat map and volcano plot (variable mRNAs) (**Supplementary Figures 3**, **4**).

The functional enrichment allowed us to understand the biological function of mRNA transcripts with high differential expression. As expected, annotations revealed several biological processes, among which we highlighted the transcriptional regulation process (*Zfpm2*, *Satb1*, and *Lef1*), cell adhesion (*Itgb1*), the immune system (*Themis*) and cellular metabolism processes as growth factor gene transcripts (*Areg* and ATP catalysts as *Lars2)* (**Supplementary Figure 5**).

**Figure 2:**
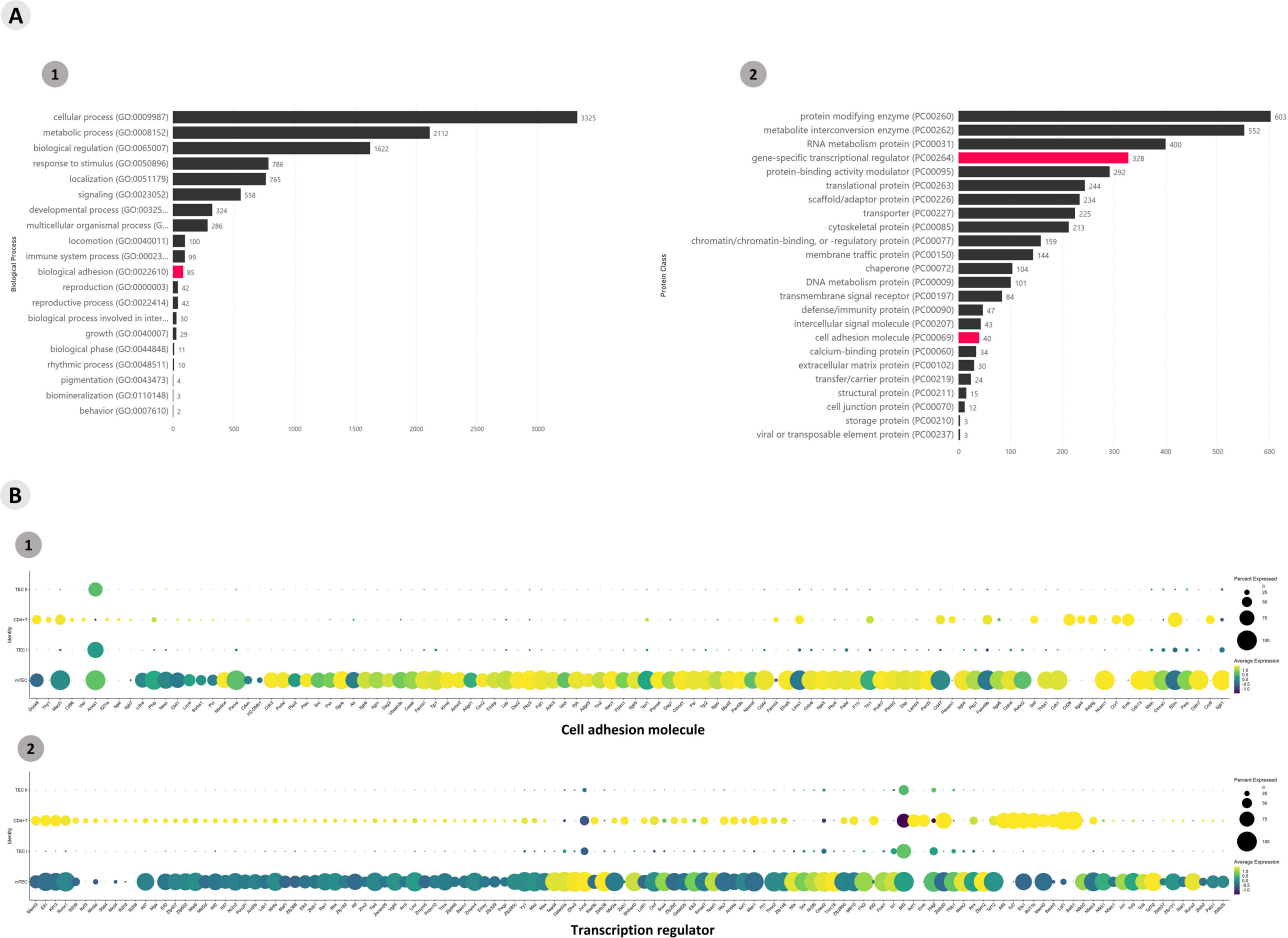
Transcriptional profiling of all mRNAs expressed among the *Aire* wild-type medullary thymic epithelial cells or wild-type naïve CD4^+^ thymocyte clusters when cocultured. **(A)** The functional annotation enrichment of all differentially expressed mRNAs enabled the identification of cell adhesion and transcriptional regulator mRNAs (A1-2); **(B)** Percent expressed and average expression of cell adhesion mRNAs or transcription regulator mRNAs among cell clusters.

Functional enrichment of all DE gene transcripts (p > 0.05) allowed the identification of mRNAs that encode transcription factors or adhesion molecules. A total of 5,675 genes were enriched according to the Panther algorithm (https://www.pantherdb.org/), and gene transcripts involved in biological and protein class processes were obtained (**Figure 2A**). A heatmap was constructed to visualize the differential expression of the mRNAs that encode adhesion molecules or transcription factor categories (**Supplementary Figure 6**). A dot-plot was drawn for the percentage of gene transcripts expressed in the cells of each cluster (**Figure 2B**).

**Figure 3:**
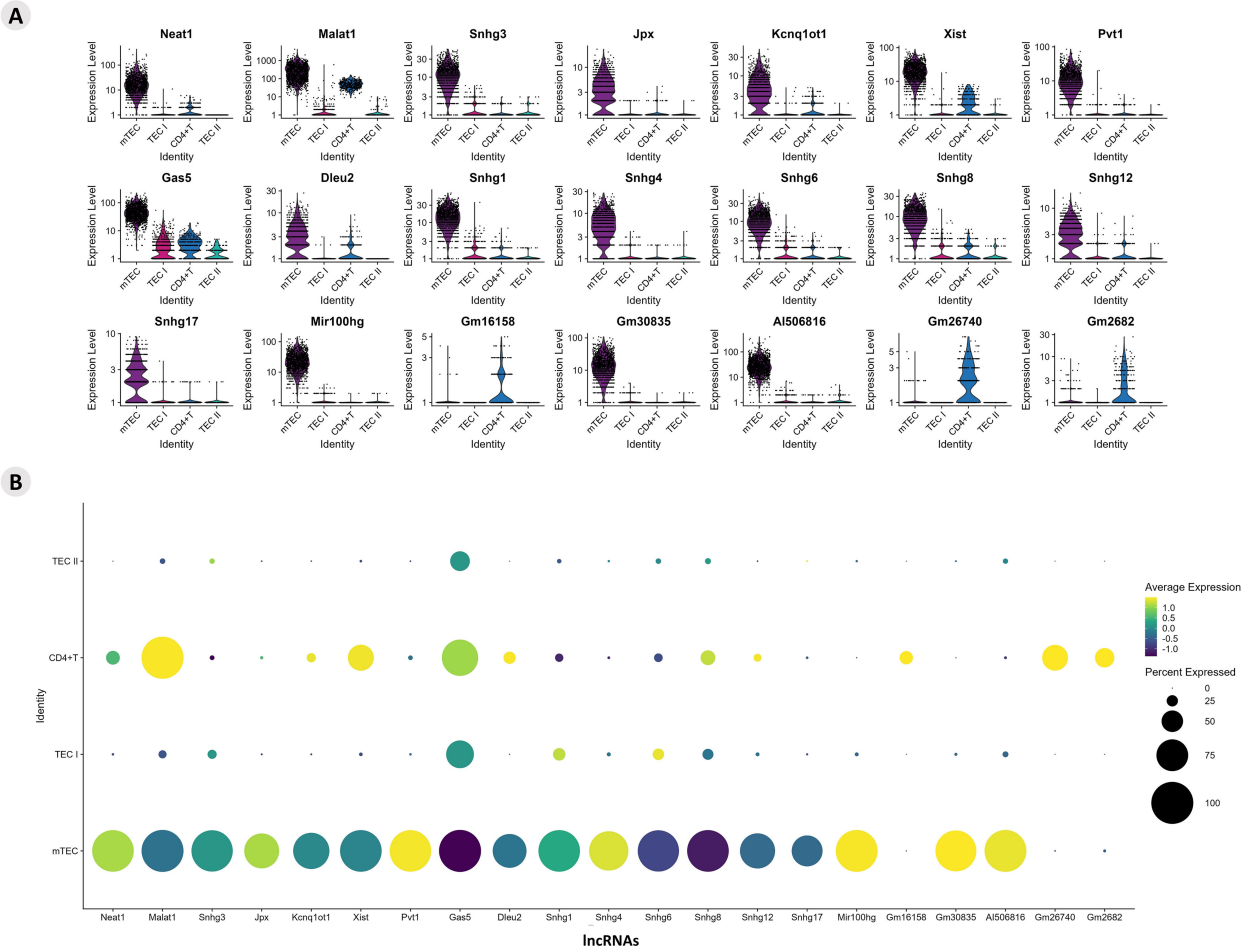
Transcriptional profiling of lncRNAs among the different *Aire* wild-type medullary thymic epithelial cells or wild-type naïve CD4^+^ thymocyte clusters. **(A)** Violin plots of percentage expression of lncRNAs **(A)**; identification of individual lncRNAs that are expressed by specific cell types; average expression of lncRNAs among the different medullary thymic epithelial cells or wild type naïve CD4^+^ thymocytes **(B)**.

The differential expression of lncRNAs was also identified, and it was possible to analyze their expression profile (**Supplementary Figure 7**). The profiles of lncRNA-type markers in the mTEC, TEC I, and TEC II clusters and in the CD4^+^ T cells are shown using violin plots (**Figure 3A**). The DE lncRNAs from this study that were already enriched and validated by other studies were identified on the LncTAR 2.0 platform (https://lnctard.bio-database.com/). The validated lncRNAs are highlighted in terms of expression level, and the percentage of lncRNAs expressed in each cluster was drawn separately in a dot-plot graph (**Figure 3B**).

**Figure 4:**
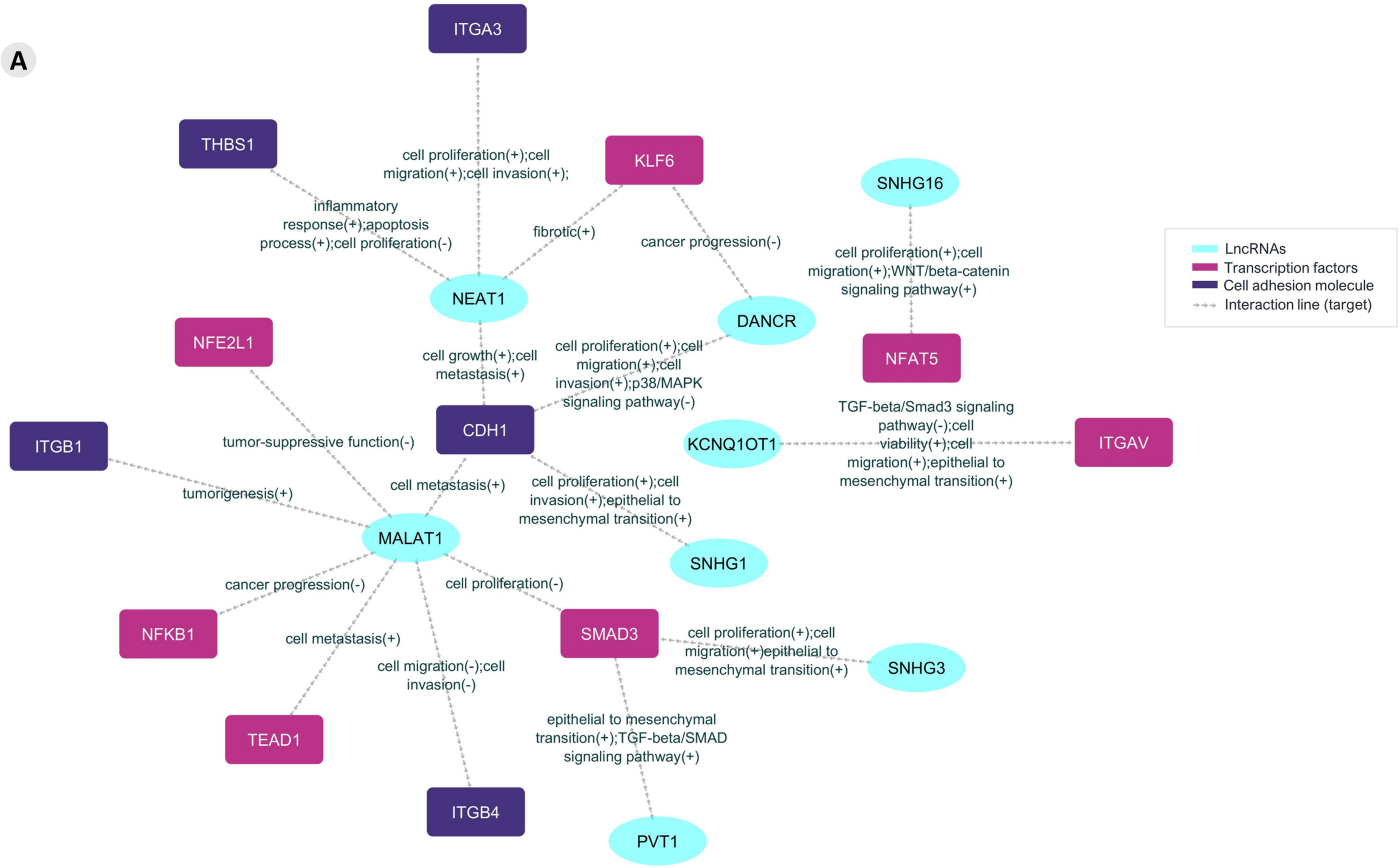
The prediction lncRNA-mRNA posttranscriptional interaction network from *Aire* wild-type mTECs and wild-type CD4^+^ thymocytes. The differentially expressed and previously validated lncRNAs observed among the *Aire* WT medullary thymic epithelial cell clusters or CD4^+^ thymocytes can establish interactions with target mRNAs that encode adhesion molecules or transcriptional regulators.

The information on those mRNAs that encode adhesion molecules or transcription factors and the previously validated lncRNAs enabled the construction of a posttranscriptional interaction network (**Figure 4**).

### AIRE protein expression

Compared to the *Aire* WT mTEC 3.10, the *Aire*-deficient (Aire ^wt/mut,^ clone CS8D6 harboring del 3554G) expressed lower amounts of the ~57 kDa AIRE protein. The remaining amount of AIRE observed in the *Aire*-deficient cells may be due to the expression of the WT allele (**Supplementary Figure 8**).

### Single-cell RNA-seq analysis of Aire-deficient clone and wild-type CD4^+^ thymocytes after adhesion

**Figure 5:**
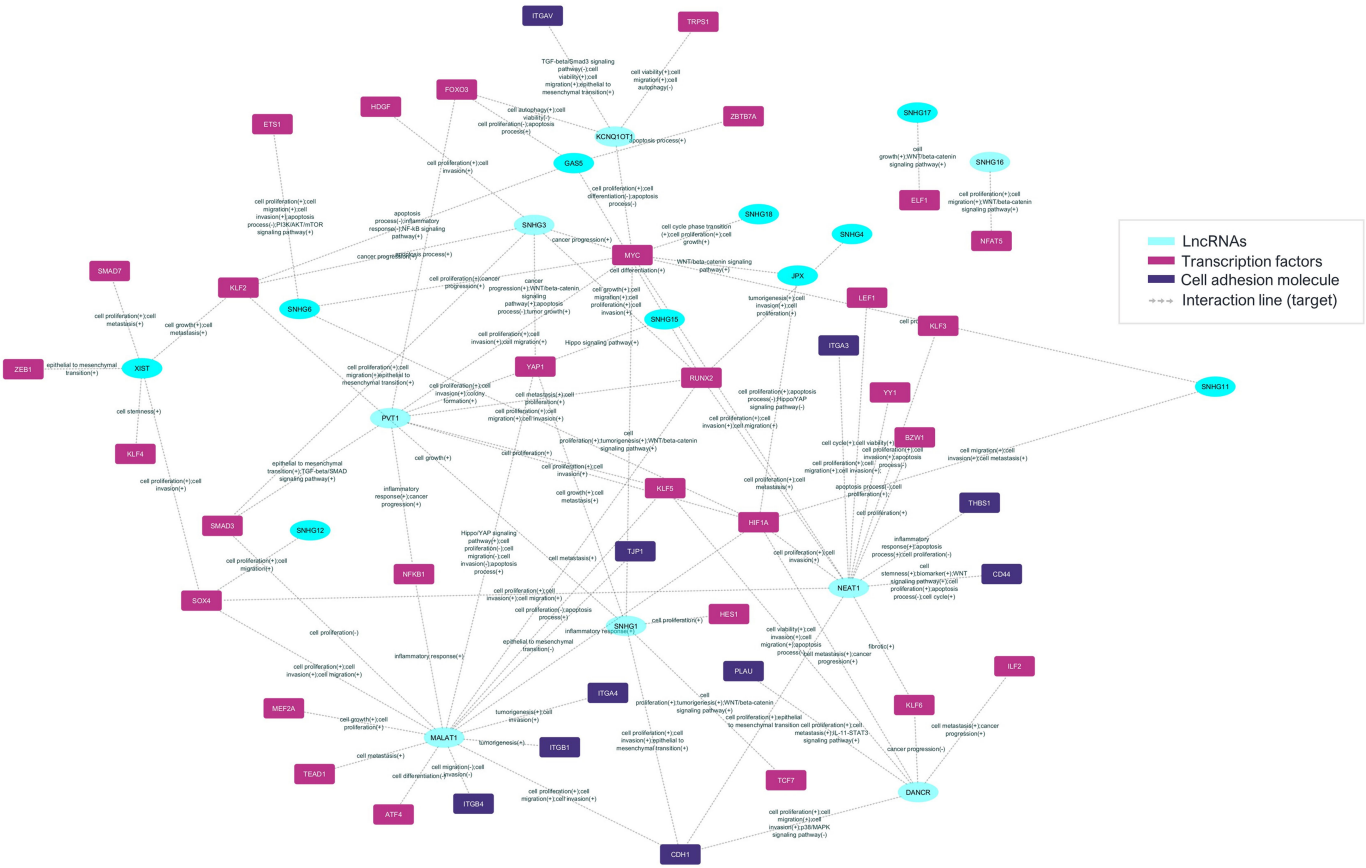
The prediction of the lncRNA-mRNA posttranscriptional interaction network between mRNAs and lncRNAs in *Aire*-deficient medullary thymic epithelial cells and wild-type CD4^+^ thymocytes. The differentially expressed and previously validated lncRNAs observed among the *Aire* mutant medullary thymic epithelial cell clusters or wild-type CD4^+^ thymocytes can establish interactions with target mRNAs that encode adhesion molecules or transcriptional regulators.

It was possible to identify lncRNAs, their target mRNA transcripts, and their respective biological processes. This information created an interaction network between lncRNAs and the respective mRNAs that encode adhesion molecules or transcription factors associated with biological function (**Figure 5**).

**Figure 6:**
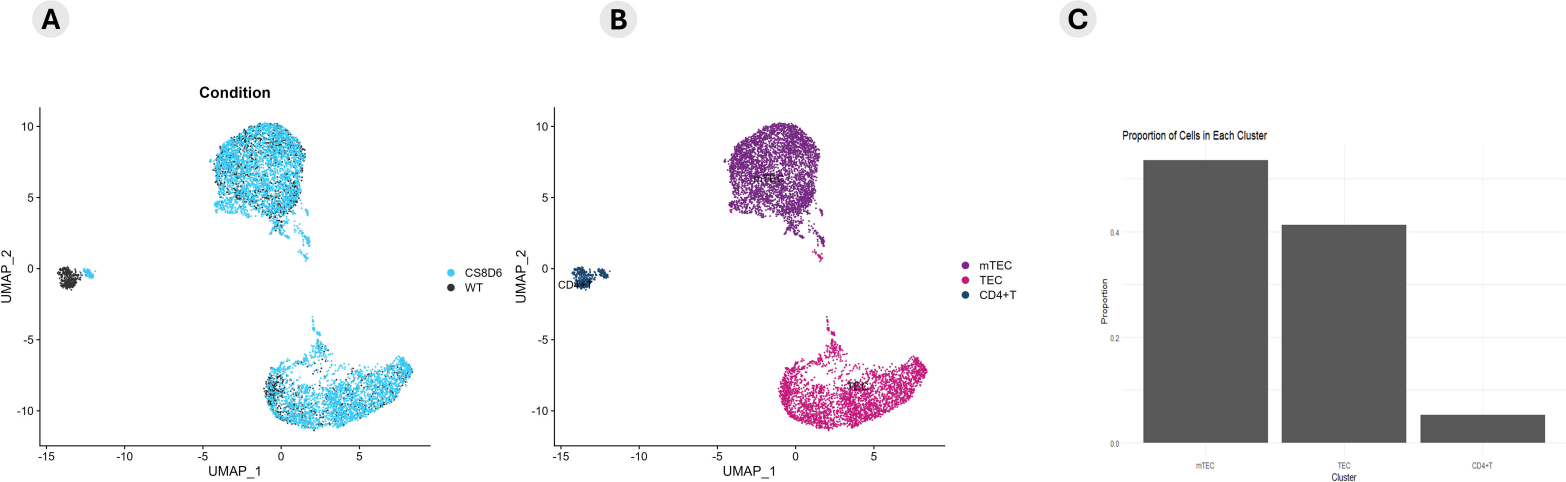
Integrative coexpression analysis. The datasets from *Aire* wild-type cells, *Aire*-deficient medullary thymic epithelial cells, and wild-type CD4^+^ thymocytes were integrated to determine how their transcriptional modulation could distinguish between *Aire* wild-type and *Aire*-deficient TECs **(A)** or between TECs, mTECs and wild-type CD4^+^ thymocytes **(B)**. A proportion plot of cells in each cluster **(C)** of the UMAP **(B)** was generated. The identification of cell clusters was performed according to the expression of specific mRNA markers (*Epcam*, *Ccna2*, and *Birc5* for TECs or mTECs) or (*Ilr7* and *Ccr7* for CD4^+^ thymocytes) **(B, C)**. The expression profiles of enriched mRNAs that encode cell adhesion molecules or transcriptional regulators are shown in the dot-plot graph (D1-2).

We subsequently investigated the integrated modulation profile of mRNA and lncRNA expression in *Aire*-deficient mTECs and WT SP CD4^+^ thymocytes. The datasets were integrated, and cellular clusters were identified using the primary markers of mTECs and CD4^+^ thymocytes (**Figure 6**).

The expression profiles of the enriched mRNAs, such as those encoding adhesion molecules or transcription factors, are shown in the dot-plot graphs (**Figure 6**). The expression levels are comparative and are represented individually for each cluster; the conditions are given on a scale between the minimum and maximum expression and the percentage of expression of these mRNAs in each cell.

**Figure 7:**
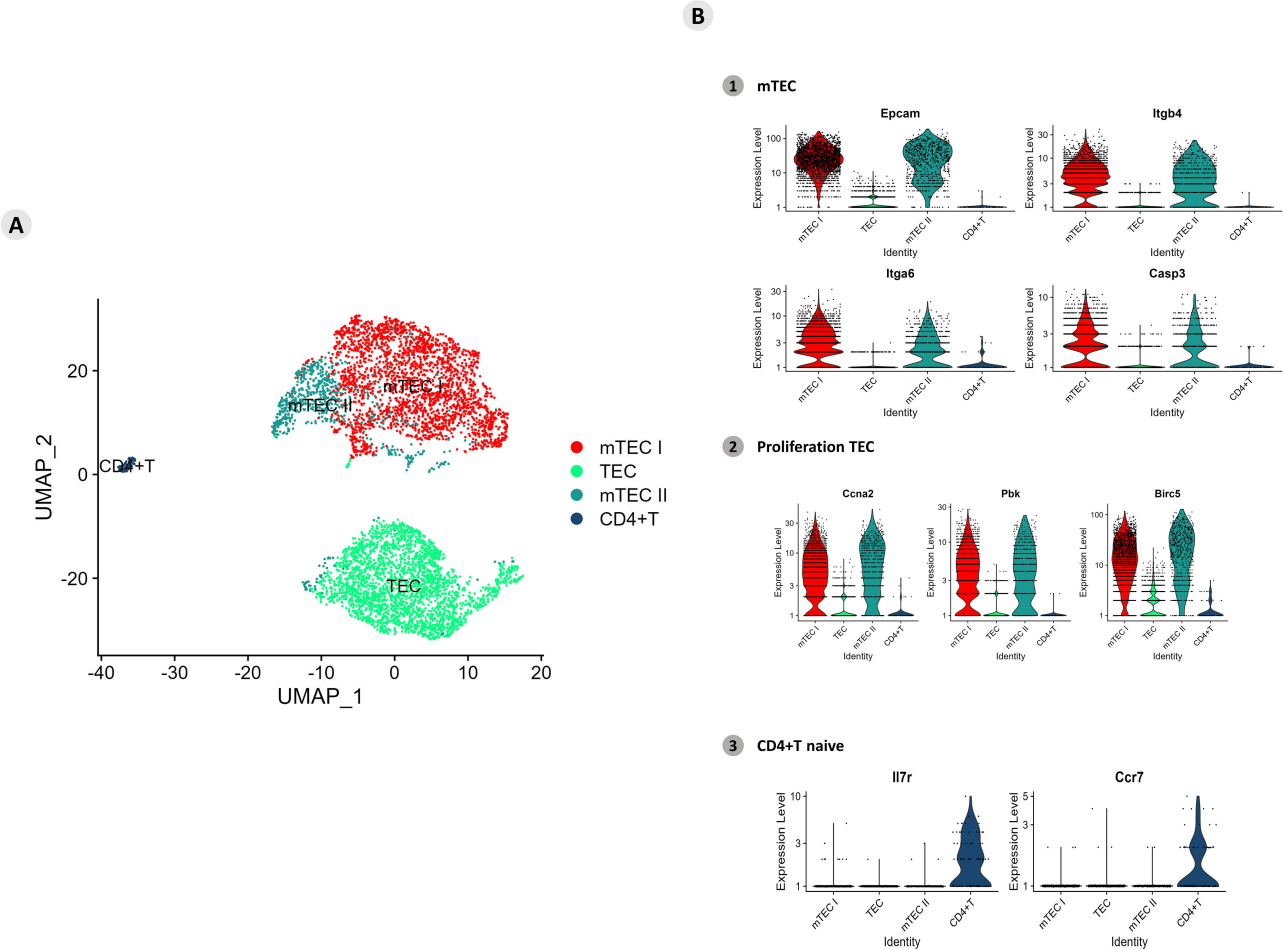
Single-cell mRNA transcriptional analysis of *Aire*-deficient medullary thymic epithelial cells cocultured with wild-type CD4^+^ thymocytes. **(A)** UMAP clustering of TECs (TEC, mTEC I, mTEC II, and CD4^+^ thymocytes) based on their transcriptional profiles. **(B)** mTECs were identified based on their expression of *Epcam*, *Itgb4*, *Itgb6* and *Casp3* mRNA markers (B1), proliferative TECs based on *Ccna2*, *Pbk* and *Birc5* expression (B2), and naïve CD4^+^ thymocytes based on *Ilr7* and *Ccr7* expression (B3).

The clusters of mTECs were identified according to the transcription of the markers (*Epcam*, *Itgb4*, *Itgb6*, and *Casp3*/Sca1) for mTECs and (*Ccna2*, *Pbk* and *Birc5*) for proliferative TECs the expression of single-positive CD4^+^ thymocyte-specific markers (*IL7r* and *Ccr7*) (**Figure 7**; **Supplementary Figure 9**).

The transcription of marker mRNAs (*Ccna2*, *Pbk*, and *Birc5*) that identify proliferative mTECs was tested, and the results revealed a unique cluster of these cell types (**Figure 7**; **Supplementary Figure 10**). The transcription of the *Il7r* and *Ccr7* markers revealed a homogeneous cluster characteristic of naïve CD4^+^ thymocytes (**Figure 7**; **Supplementary Figure 11**).

The mRNA transcripts with high differential expression are highlighted and shown in the heatmap (top mRNAs). The mRNA transcripts with the most significant variability are highlighted in the heat map and volcano plot (variable mRNAs) (**Supplementary Figures 12**, **13**). The functional annotation revealed several biological processes, among which transcriptional regulation, cell adhesion, the immune system, and other cellular metabolism processes were highlighted (**Supplementary Figure 14**).

**Figure 8:**
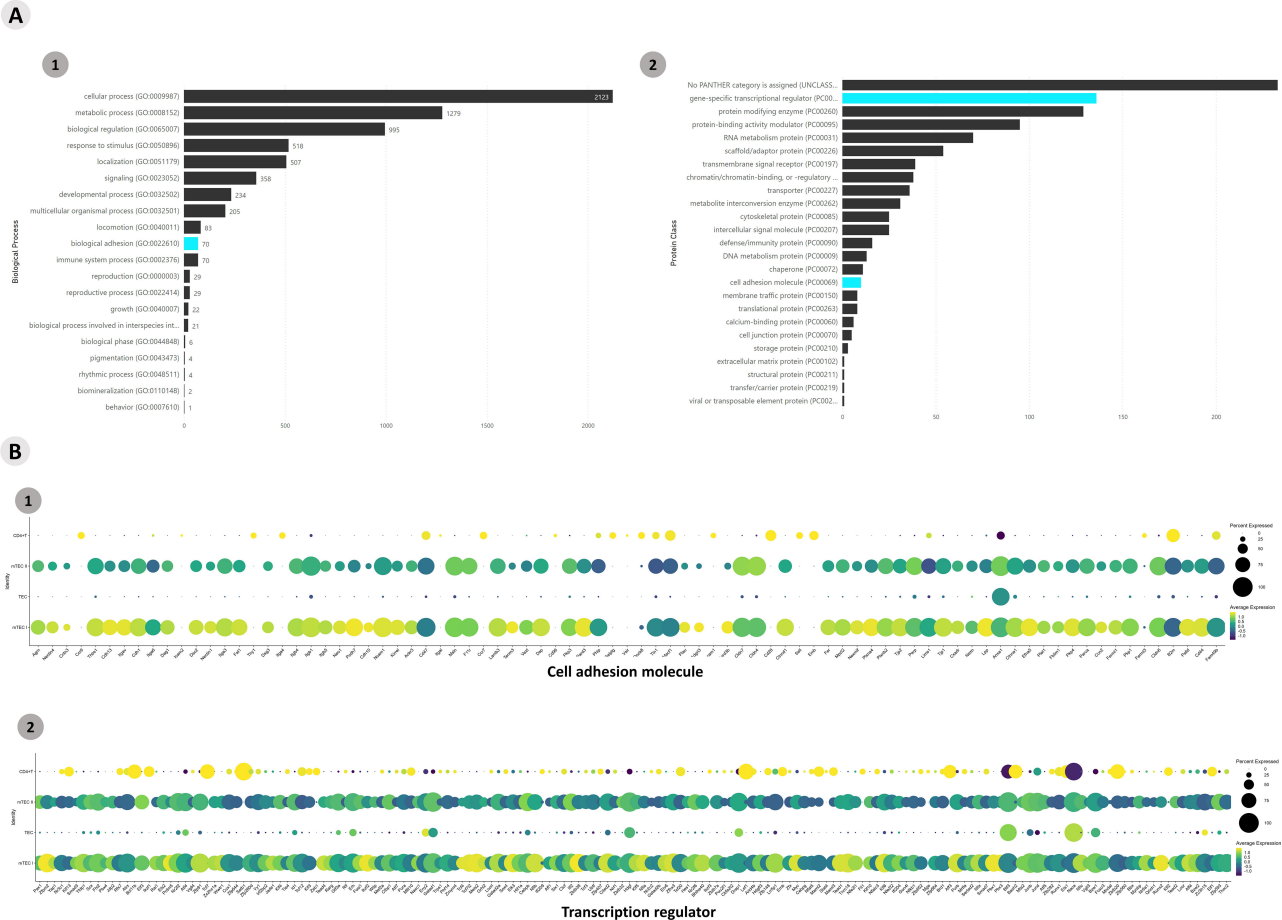
Transcriptional profiling of all mRNAs expressed among the different *Aire*-deficient medullary thymic epithelial cells or wild-type CD4^+^ thymocyte clusters when cocultured. **(A)** The functional annotation enrichment of all differentially expressed mRNAs enabled the identification of cell adhesion and transcriptional regulator mRNAs (A1-2); Percent expressed and average expression of cell adhesion mRNAs **(B1)** or transcription regulator mRNAs among cell clusters **(B2)**.

Functional enrichment of all DE mRNA transcripts (p > 0.05) was performed with the (The Gene Ontology Resource, https://www.geneontology.org/) and mRNAs that encode transcription factors or adhesion molecules were identified and selected. A total of 3,538 genes were enriched in the Panther, and gene transcripts involved in “biological process” and “protein class” processes were obtained (**Figure 8**; **Supplementary Figure 15**). The *Aire* WT and *Aire*-deficient (CS8D6) datasets were integrated to combine shared statistically significant mRNA expression values characterizing different cell types. The identification of DE lncRNAs allowed us to assess the individual expression level of each lncRNA.

**Figure 9:**
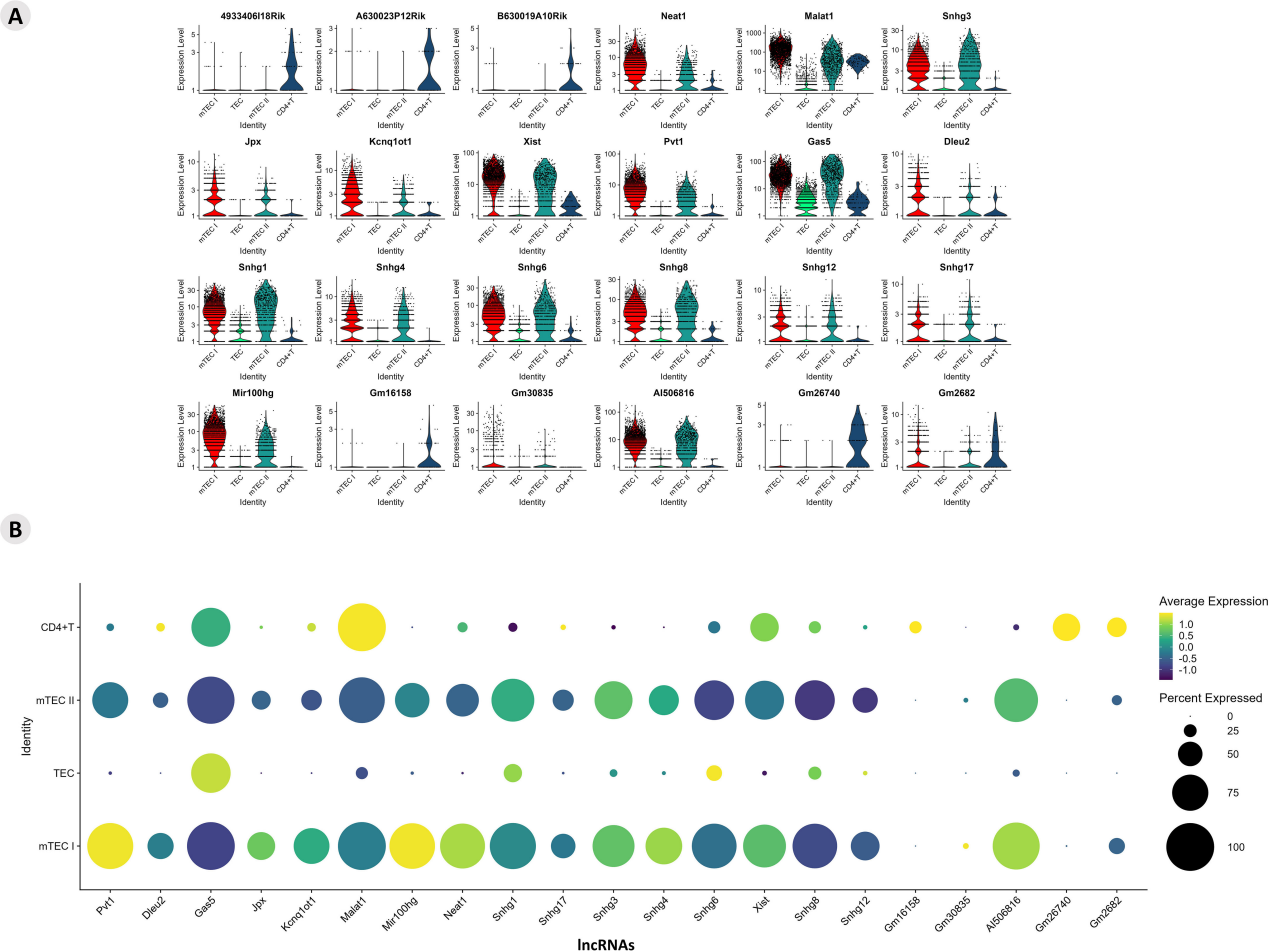
Transcriptional profiling of lncRNAs among the different *Aire*-deficient medullary thymic epithelial cells or wild-type CD4^+^ thymocyte clusters when cocultured. **(A)** Percentage expression and average expression of lncRNAs **(A)**; identification of individual lncRNAs that are expressed by specific cell types **(B)**; average expression of lncRNAs among the different medullary thymic epithelial cells or wild type naïve CD4^+^ thymocytes.

The profiles of lncRNA-type markers in mTEC I, mTEC II, and WT single-positive CD4^+^ thymocyte populations and TEC clusters are shown with violin plots (**Figure 9A**). These lncRNAs are highlighted in terms of their expression levels, and the percentages of lncRNAs expressed in each cluster are shown separately in the dot-plot graphs (**Figure 9B**). The TEC, mTEC I, mTEC II, and SP CD4^+^ thymocyte clusters expressed the previously validated (LncTAR 2.0 platform) lncRNAs, Neat1, Snhg16, Dancr, Kcnq1ot1, Malat1, Snhg1, Snhg3, and Pvt1.

Results suggest that these lncRNAs might be influenced by *Aire* in mTECs since its expression is differential when comparing *Aire* WT versus *Aire*-deficient cells. In addition, the lncRNA-mRNA interaction networks allowed us to observe that these lncRNAs exert posttranscriptional control on the following transcription factor mRNAs *Klf6*, *Nfe2l1*, *Nfat5*, *Itgav*, *Nfkb1*, *Smadd3* and *Tead1* and the adhesion molecule mRNAs *Itga3*, *Thbs1*, *Itgb1*, *Cdh1* and *Itgb4* (**Figures 4**, **5**).

### Integrative coexpression analysis

**Figure 10:**
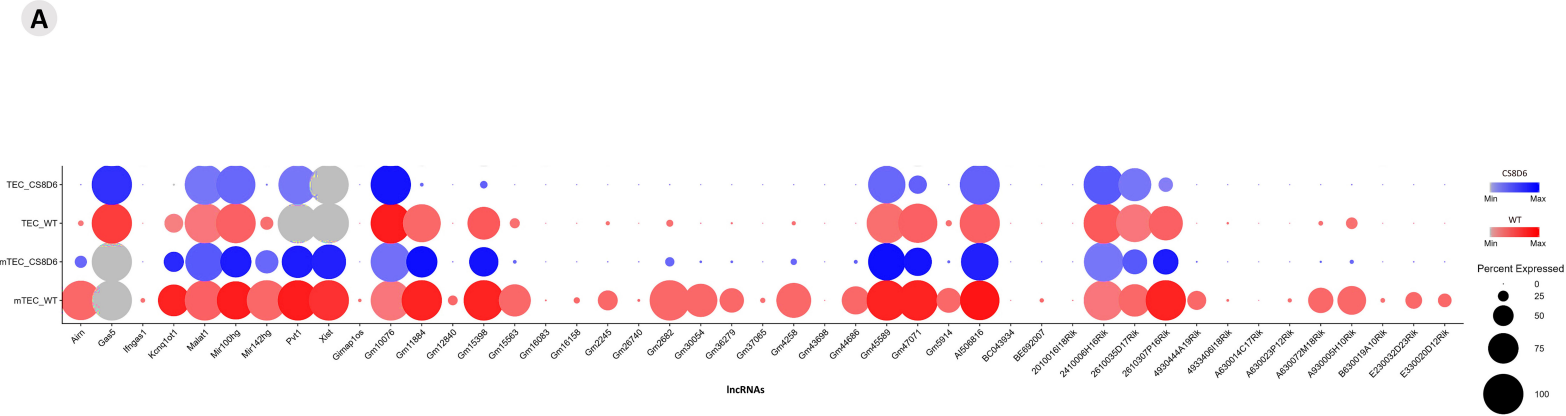
Integrated analysis of lncRNA expression among the cell types. The comparative expression levels of each lncRNA are individually represented for each group or condition (*Aire* WT or *Aire*-deficient), and the relative percentage of each TEC type is shown.

Using integrative analysis, we also identified the expression profiles of the lncRNAs (**Figure 10**). The expression levels are comparative and are represented individually for each cell type; the conditions are given on a scale between the minimum and maximum expression and the percentage of expression of these lncRNAs in each cell.

**Figure 11:**
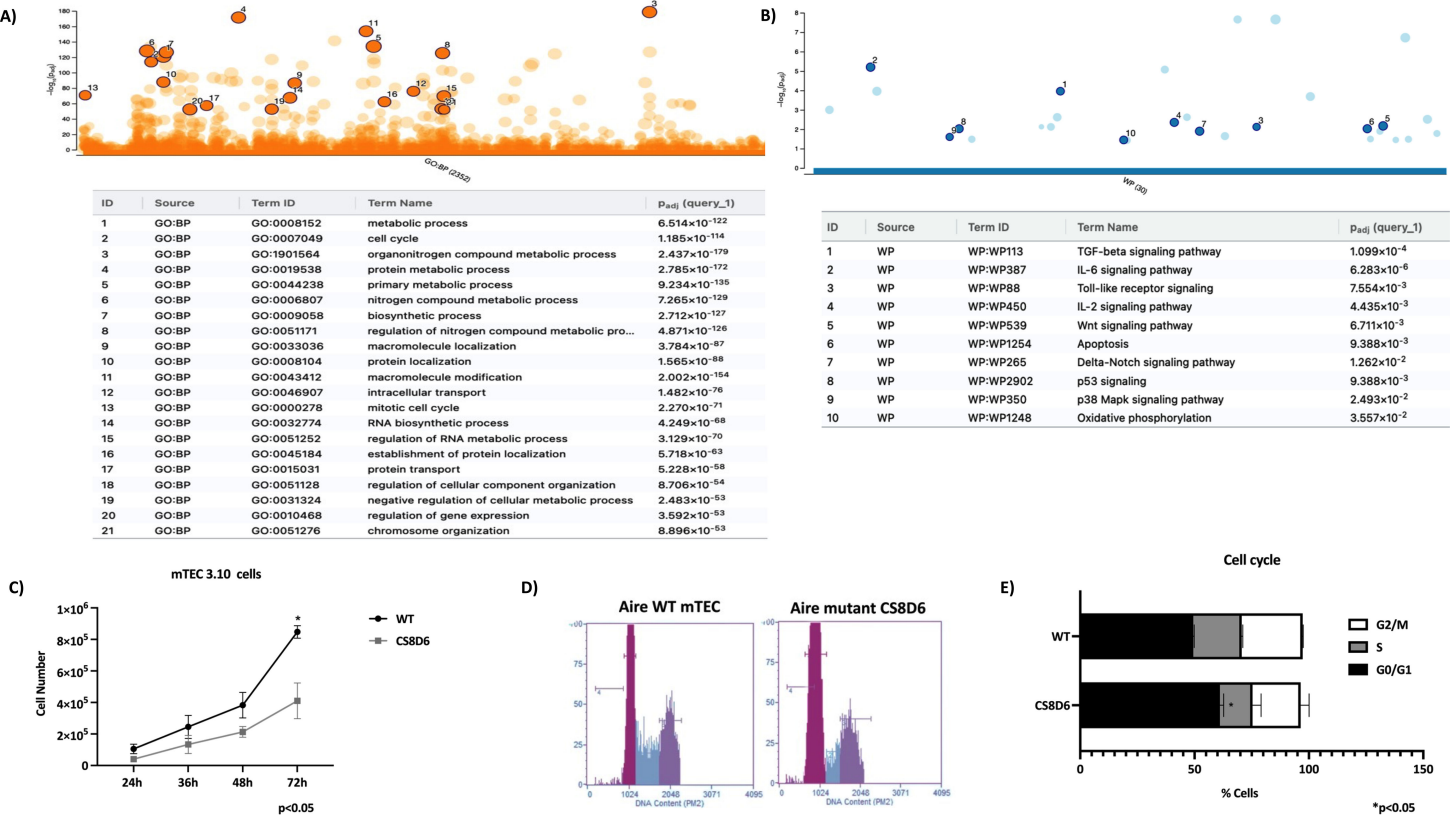
Cell cycle analysis of *Aire* wild-type and *Aire*-deficient mTECs. Manhattan plots showing the biological pathways associated with *Aire* wild-type and *Aire*-deficient mTECs, including the mitotic cell cycle, *Tgf beta*, *IL-6*, *Wnt*, apoptosis, and Notch signaling pathways **(A, B)**. Growth and cell cycle analysis comparing *Aire* wild-type mTECs with *Aire*-deficient mTECs; percentage of mTECs in the different phases of the cell cycle **(C-E)**. Data are presented as mean ± s.d. (replicates n = 3). The differences were evaluated using Student *t* test (p values are indicated for each comparison when pertinent).

The expression profiling also revealed the modulation of genes involved in the mitotic cell cycle, the *Tgf beta*, *Il-6*, *Wnt*, apoptotic, and Notch signaling pathways. Given this, we asked whether *Aire* could influence mTEC growth and the cell cycle. *Aire* deficiency impaired the growth of the CS8D6 mutant clone, which remained in the G0/G1 cell cycle phase more than its *Aire* WT counterpart did (**Figure 11**).

**Figure 12:**
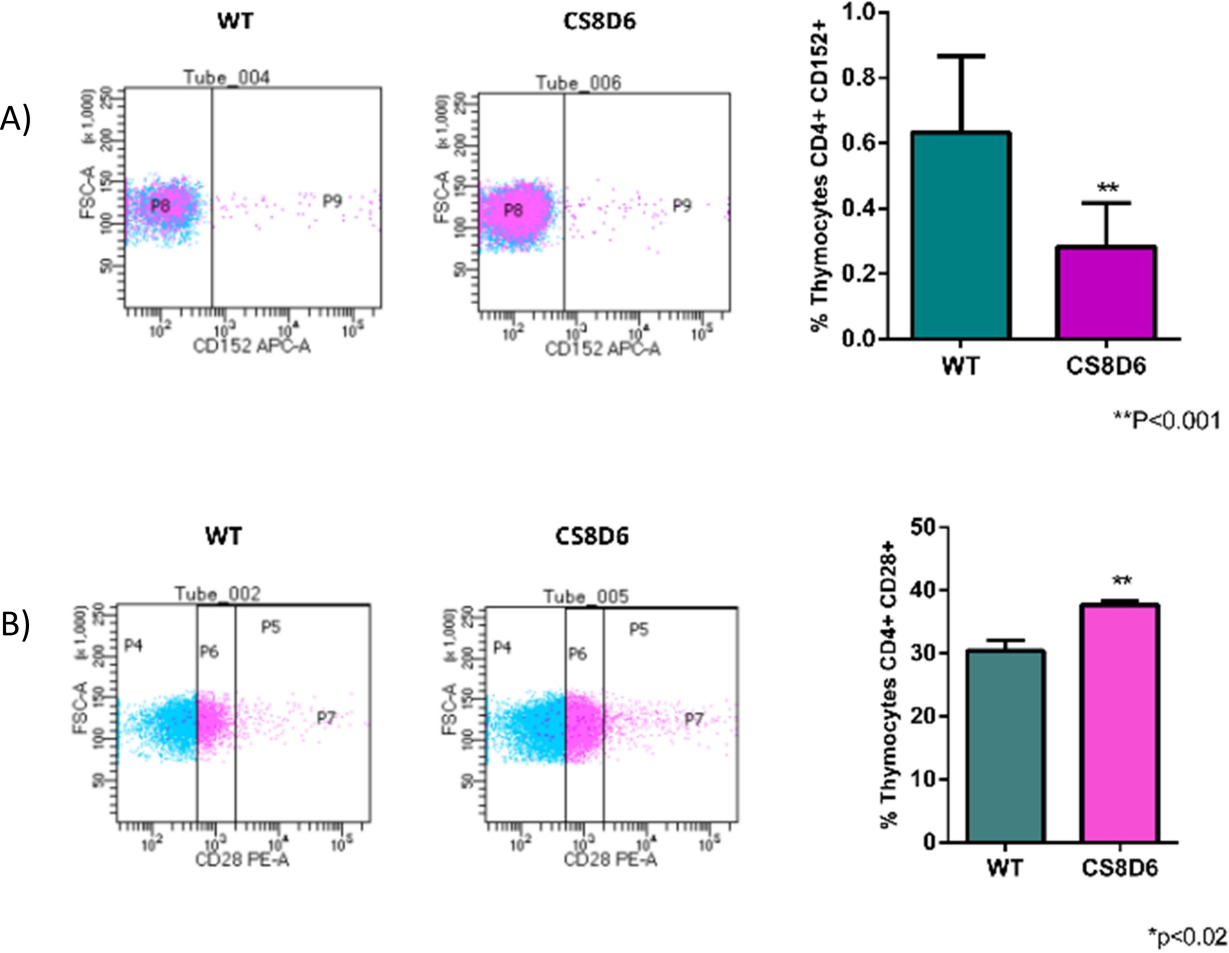
Expression levels of surface proteins in wild-type CD4^+^ thymocytes cocultured with wild-type or *Aire*-deficient mTECs. The expression of CD152 **(A)** or CD28 **(B)**. Data are presented as mean ± s.d. (replicates n = 3). The differences were evaluated using Student *t* test (p values are indicated for each comparison when pertinent). (**) = statistical significance.

We subsequently investigated whether *Aire* deficiency in mTECs could affect the expression of molecules associated with T-cell activation in adhered thymocytes. Wild-type CD4^+^ thymocytes that adhered to *Aire*-deficient mTECs downregulated CD152 (CTLA4) and upregulated CD28 (**Figure 12**).

**Figure 13:**
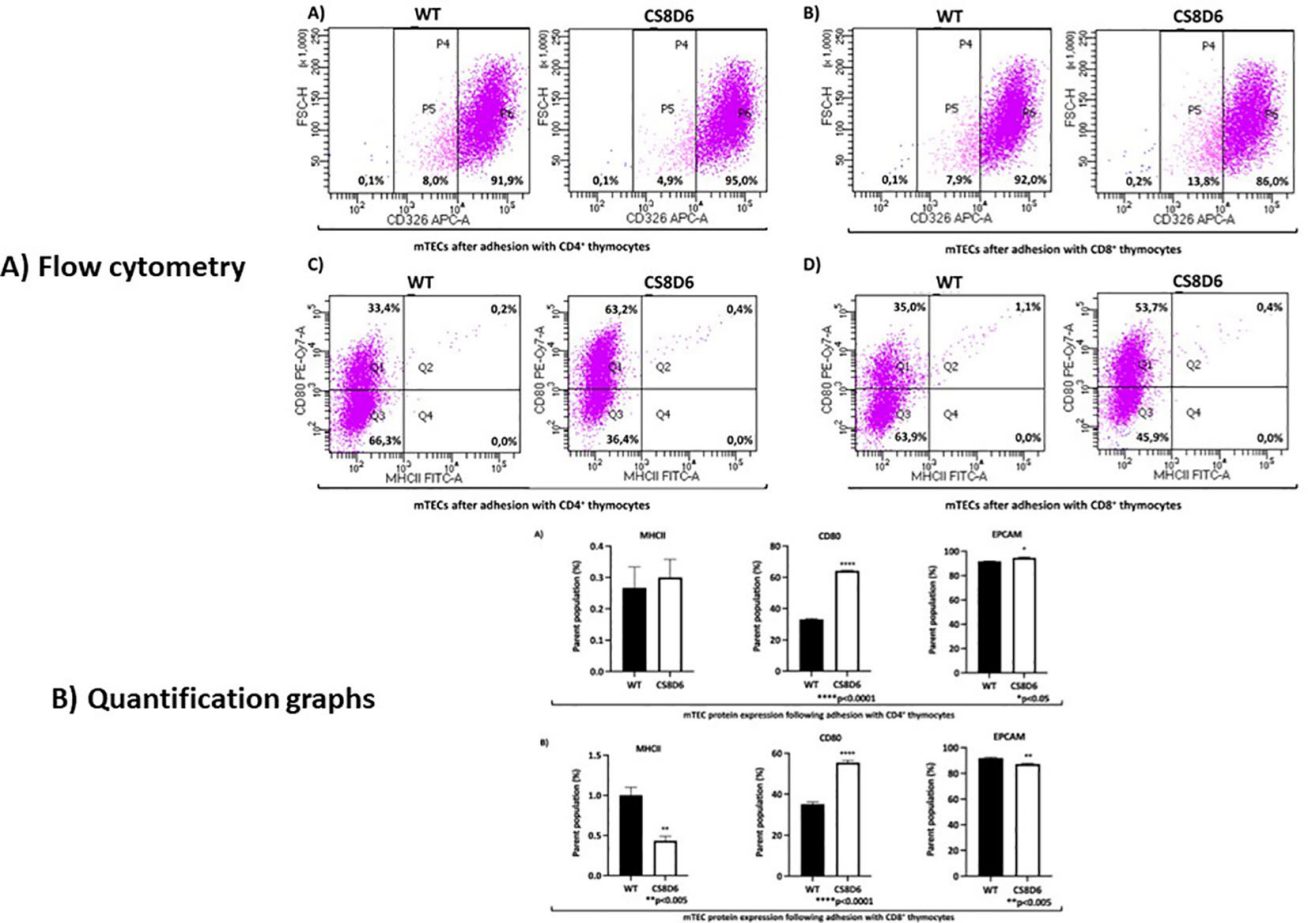
The expression levels of surface proteins in Aire wild-type or *Aire*-deficient mTECs cocultured with wild-type CD8^+^ or CD4^+^ thymocytes. CD326 (EPCAM) expression increased when mTECs were cocultured with CD4^+^ cells and decreased when mTECs were cocultured with CD8^+^ thymocytes **(A, B)**. The percentage of double-positive CD80^+^/MHC-II^+^ mTECs increased when they adhered to CD4^+^ cells and decreased when they adhered to CD8^+^ thymocytes **(C, D)**. Data are presented as mean ± s.d. (replicates n = 3). The differences were evaluated using Student *t* test (p values are indicated for each comparison when pertinent). (*), (**), (***) = statistical significance.

Additionally, CD326 (EPCAM) protein expression slightly increased in *Aire*-deficient mTECs when they adhered to SP CD4^+^ cells and decreased when they adhered to CD8^+^ thymocytes, and the number of double-positive CD80^+^/MHC-II^+^ mTECs slightly increased when they adhered to SP CD4^+^ cells and decreased when they adhered to CD8^+^ thymocytes (**Figure 13**).

## Discussion

In this study, we explored the upstream effect of the autoimmune regulator (*Aire*) gene on the transcriptional and posttranscriptional control exerted by long noncoding RNAs (lncRNAs) when medullary thymic epithelial cells (mTECs) and wild-type (WT) single-positive (SP) CD4^+^ thymocytes were cocultured. We showed that the mRNAs controlled by *Aire* in mTECs, including those that encode transcription factors, such as *Klf6*, *Nfe2l1*, *Nfat5*, *Itgav*, *Nfkb1*, *Smadd3*, and *Tead1*, or cell adhesion molecules, for example, *Epcam*, *Itga3*, *Thbs1*, *Itgb1*, *Cdh1* and *Itgb4*, are regulated by lncRNAs, such as Neat1, Snhg16, Dancr, Kcnq1ot1, Malat1, Snhg1, Snhg3 and Pvt1. Single-cell transcriptome analysis of *Aire* WT or *Aire-*deficient mTECs cocultured with WT SP CD4^+^ thymocytes followed by interaction network analysis revealed the intricate posttranscriptional control exerted by lncRNAs, which are controlled by *Aire* and the cell adhesion process.

Thymic crosstalk must occur for autoreactive thymocytes to undergo negative selection (3, 8, 46). mTEC–thymocyte adhesion is crucial in this process, as mTECs present peripheral tissue autoantigens (PTAs) to SP CD4^+^ or CD8^+^ thymocytes, and thymocyte clones expressing TCRα/β that avidly recognize PTAs may be eliminated by apoptosis. The *Aire* gene controls PTA and adhesion molecules in mTECs; *Aire* and cell adhesion may act in combination since *Aire* can control adhesion, and adhesion regulates *Aire* (18, 31).

Our group has studied the effect of the *Aire* gene on the expression of mRNAs that encode PTAs or cell adhesion molecules and noncoding RNAs (miRNAs or lncRNAs) in mTECs (18, 28, 31). Using an anti-*Aire* small interfering RNA (siRNA), we observed that decreasing *Aire* expression could affect the transcriptome and biological function of mTECs (19). Therefore, we gathered evidence that a decrease in *Aire* expression, but not its complete abrogation, is enough to disarrange the mTEC transcriptome.

This finding is in line with the findings of APS-1 syndrome patients who are heterozygous for AIRE gene mutations in the AIRE protein SAND domain, which are dominant mutations (47–50); that is, many APS-1 patients only have one WT AIRE allele; therefore, the expression of the normal version of the AIRE gene is reduced but not absent.

Accordingly, in this study, we chose to use a heterozygous clone of mTEC cells in which one of the *Aire* alleles was knocked out through the CRISPR-Cas9 system (*Aire*-deficient, clone CS8D6 *Aire* ^wt/mut^) (40) as a model system to assess the effect of partial *Aire* abrogation on the transcriptome of these cells. Moreover, we used the single-cell RNA-seq method followed by detailed data analysis, which revealed that the cultured mTEC 3.10 cell line is composed of individual groups of TECs that differ in their transcriptional profile, including resting and proliferative mTECs that express the mRNA markers *Ccna2*, *Pbk*, and *Birc5*.

In a previous study, our group reported that *Aire* could control lncRNAs in mTECs (18). However, information on whether these RNA species participate in the gene expression control of mTECs is still lacking. Considering that i) among the mechanisms of control involving lncRNAs, the probability of mRNA-lncRNA posttranscriptional interactions occurring is high (51), ii) transcription factors represent one of the most essential classes of transcriptional controllers in mammalian cells and can be controlled through networking with other molecules (52), and iii) cell adhesion molecules are crucial for thymic crosstalk (8), we investigated whether lncRNAs may control the mRNAs that encode transcription factors or cell adhesion molecules in mTECs. Indeed, we established for the first time posttranscriptional interaction networks involving mRNAs encoding transcription factors or adhesion molecules with lncRNAs. Therefore, we assessed the different groups of mTECs that adhere to thymocytes based on transcriptome expression and control, which are initially generated by *Aire*, a transcriptional modulator; subsequently, by classical transcription factors; and finally, by lncRNAs.

To further understand the intricate interactions among these RNA species, we initially used single-cell RNA-seq analyses. Accordingly, *Aire* WT mTECs (mTEC 3.10) that adhere to WT SP CD4^+^ thymocytes exhibit three clusters according to their transcriptional profile: the TEC I, TEC II, and mTEC clusters. The mTEC cluster comprises the majority of the cell line and expresses the classical *Epcam*, *Itgb4*, *Itga6*, and *Casp3* transcriptional gene markers. In addition, the expression of *Ccna2*, *Pbk*, and *Birc5* suggested that this cluster is composed of proliferative mTECs. Otherwise, the cluster of WT SP CD4^+^ thymocytes remained homogeneous and expressed the *Il7r* and *Ccr7* transcriptional gene markers, indicative of their naïve phenotype.

Given that i) the cell-cell adhesion process activates the expression of a broad set of mRNAs in mammalian cells (52, 53) and ii) mTEC cell adhesion regulates mRNAs that encode transcription factors and lncRNAs (18), we investigated the broad range of adhesion molecules and transcription factor mRNAs that were modulated in WT and *Aire*-deficient mTECs and their posttranscriptional interactions with lncRNAs.

It is conceivable that during the adhesion process of mTECs with thymocytes, there are differences between mTEC-thymocyte pairs in terms of adhesion strength between cells. If this occurs, it should cause differences in the transcriptional responses of the cells involved. Given our experiment’s complexity, we did not address this plausible issue in this work, but this limitation does not invalidate the results obtained.

Clustering of *Aire*-deficient mTECs cultured with WT SP CD4^+^ thymocytes was performed and plotted on a UMAP plot. Three clusters were identified for the TECs (TECs, mTEC I, and mTEC II), which differed from the clusters formed with *Aire* WT mTECs. Although they are distinct clusters, mTEC I and mTEC II cells expressed *Epcam*, *Itgb4*, *Itga6*, and *Casp3*, as well as the proliferation-related genes *Ccna2*, *Pbk*, and *Birc5*, suggesting that proliferative mTECs form these clusters. The TEC cluster comprised an important fraction of the *Aire*-deficient clone and differed from the WT according to the lack of expression of mTECs and proliferation markers. Like what was observed in the WT coculture, the SP CD4^+^ thymocyte cluster remained homogeneous; however, the expression of the naïve *Ilr7* and *Ccr7* transcriptional markers was discrete.

The single-cell analysis in this study allowed us to show how heterogeneous an mTEC population is in terms of its transcriptome. The mTECs studied here, both *Aire*-WT and *Aire*-deficient, are genetic clones in concept, as they originate from the mitotic division of a single cell. However, analysis of the single-cell transcriptome showed that transcriptional gene expression in these cells was not uniform, which gave them distinct clusters, mTECs, TEC I and TEC II, whose transcriptional profiles are influenced by *Aire*. The differences in the transcriptomic profiles of mTECs should affect the representation of self-antigens by each of the clusters.

This study was performed only with mTEC cells in coculture with thymocytes. The rationale for this protocol is that mTECs are in constant contact with thymocytes in the thymus. However, this made it impossible to know whether the heterogeneity of mTECs is an intrinsic characteristic of these cells or whether this happens because of contact with thymocytes, which could be a limitation of the study. Anyway, coculture more closely reproduces the physiology of the thymus.

In addition, we observed that the TEC, mTEC I, mTEC II, and SP CD4^+^ thymocyte clusters expressed the transcription factor mRNAs *Klf6*, *Nfe2l1*, *Nfat5*, *Itgav*, *Nfkb1*, *Smadd3* and *Tead1* and the adhesion molecule mRNAs *Itga3*, *Thbs1*, *Itgb1*, *Cdh1* and *Itgb4*, indicating that these mRNAs are coexpressed in TECs and thymocytes. Furthermore, we observed that various previously validated lncRNAs, Neat1, Snhg16, Dancr, Kcnq1ot1, Malat1, Snhg1, Snhg3, and Pvt1 (LncTAR 2.0 platform), are also under the influence of *Aire* and subsequently exert posttranscriptional control of the above mRNAs.

The post-transcriptional control of gene expression in mTEC cells has yet to be explored, but this type of control is no less critical. Instead, it represents the fine-tuning of gene expression (20). In previous work by our group (19), we observed that the downregulation of Aire in mTECs causes the expression of PTA mRNAs to be more regulated by miRNAs. The same type of control occurred in the present study, which involved the analysis of lncRNAs, transcription factor and cell adhesion mRNAs. Aire WT mTECs showed eight lncRNAs that exert control on seven transcription factor mRNAs and five cell adhesion molecule mRNAs. Aire-deficient mTECs (clone CS8D6) showed the participation of 18 lncRNAs that act on 30 transcription factor mRNAs and ten cell adhesion molecule mRNAs. Aire-deficient mTECs showed a more significant number of mRNA-lncRNA interactions. Since lncRNAs can exert negative control over mRNAs by blocking their translation into proteins (20), the result obtained in this study discovers a new mechanism for how Aire deficiency in mTECs can impact gene expression control, using lncRNAs as intermediaries.

The data were integrated and reanalyzed to verify how robust the datasets are and whether they could distinguish between *Aire* WT, *Aire*-deficient mutant mTECs, and WT SP CD4^+^ thymocytes. In terms of adhesion molecule mRNAs, we highlight *Adgrl3*, *Fermt3*, *Itgb7*, and *Pard3b*, which were preferentially expressed in *Aire* WT mTECs, and can therefore be considered markers that distinguish this type of cell. On the other hand, the expression of *Anxa1*, *B2m*, *Ccn2*, *Cdh10*, *Cldn4*, *Itgb1*, *Msln*, *Ncam1*, *Plet1*, *Plxna4*, *Tenm3*, and *Thbs1* was shared by *Aire* WT mTECs, *Aire*-deficient mTECs, and WT SP CD4^+^ thymocytes before and after adhesion. Whether, on the one hand, these markers were not accurate enough to distinguish the different cell types, the results suggest that the proteins encoded by the mRNAs certainly play crucial roles in mTEC-thymocyte adhesion.

As for the mRNAs that encode transcriptional controllers, the majority of the repertoire set was shared among all types of cells analyzed, suggesting their crucial function, highlighting *Junb*, *Pbx1*, *Runx1*, *Sox4*, *Tcf12*, *Tnrcc18*, *Zbtb20*, *Zfhx3* and *Zfpm2*. However, the mRNAs *Aff3* and *Ikzf3* were distinguishers of SP CD4^+^ thymocytes, and *Ifi203* distinguished well from the population of *Aire* WT mTECs.

As for lncRNAs, we highlight lfngas1, Gimap1os, Gm15563, Gm26740, Bc044934, BE692007, A630023P12Rik and B630019s10Rik as distinguishers of SP CD4^+^ thymocytes due to their preferential expression in this cell type. Among the set of lncRNAs noted, Airn, Gas5, Malat1, Mir100hg, mir142hg, Pvt1, Xist, Gm10076, Gm11884, Gm45589, Gm47071, AI506816, 2410006H16Rik, 2610035D17Rik and 2610307P16Rik were coexpressed among *Aire* WT and *Aire*-deficient mTECs and SP CD4^+^ thymocytes, suggesting that they have an essential function in the different cell types analyzed. The lncRNAs Ifngas1, Gimap10s, BC043934, BE692007, A630014C17Rik, Gm16083 and Gm43698 were distinguishers of SP CD4^+^ thymocytes due to their preferential expression in this type of cell. These lncRNAs may be associated with the functional profile of naïve CD4^+^ thymocytes.

Therefore, we investigated the functional consequences of *Aire* gene deficiency on mTEC–thymocyte adhesion. By reanalyzing the transcription profiles of the mRNAs, we observed that the expression of those mRNAs that encode proteins involved in the cell cycle was altered. In this way, we tested the cell cycle distribution of mTECs and confirmed that this biological function is affected when *Aire* is downregulated. *In vivo*, cell cycle disturbance in mTECs certainly contributes to disruption of the thymic medulla, as observed in *Aire*-deficient mice (53, 54).

*Aire* deficiency in mTECs was shown to influence the expression of CD4^+^ thymocyte surface markers during mTEC–thymocyte adhesion. The surface proteins CD152 (CTLA4), which are implicated in cell signaling (55, 56), and CD28, which are involved in T-cell proliferation and survival, cytokine production, and T-helper type-2 development and compete with CTLA4 (57, 58), were disrupted.

Furthermore, the levels of CD326 (EPCAM), which is a crucial adhesion protein; CD80, which binds to CD28 or CD152 (CTLA-4) (59, 60) on thymocytes; and MHC-II, which presents self-antigens to thymocytes, were also modulated when mTECs adhered to SP CD4^+^ or CD8^+^ thymocytes.

Others have investigated the expression of CTLA-4 in mTECs, showing that this marker is influenced by *Aire* (61) or that in the absence of *Aire*, mTECs abnormally express CTLA-4 (62). In our study, we have demonstrated a new aspect of this relationship. We found that SP CD4^+^ or CD8^+^ thymocytes, when adhered to *Aire*-deficient mTECs, downregulate CTLA-4.

This finding not only adds to the existing body of knowledge but also underscores the broad functional impact of decreasing *Aire* on mTECs, which are crucial in central tolerance induction by mimicking the different tissues and organs of the body (63). In summary, our study highlights the importance of *Aire* that is expressed in mTECs in regulating CTLA-4 expression in adhered thymocytes.

## Conclusion

The adhesion of mTECs to thymocytes triggers complex control of the expression of transcription factor mRNAs and downstream mRNAs involved in cell adhesion in both types of cells. Additionally, cell-cell adhesion favors the expression of lncRNAs that act as posttranscriptional controllers of mRNAs that encode transcription factors or adhesion molecules. The interaction networks involving mRNA and lncRNAs demonstrate the possibility that *Aire* deficiency increases the participation of lncRNAs that regulate mRNAs.

Single-cell RNA-seq analysis revealed the complexity of the transcriptional differences between mTECs and naïve SP CD4^+^ thymocytes, which are the basis of immunological tolerance. *Aire* deficiency in mTECs was shown to be pervasive, influencing the transcriptomes of mTECs and SP CD4^+^ adherent thymocytes.

## Data Availability

The data presented in the study are deposited in the NCBI BioProject repository, accession number PRJNA1001046.

## Supplementary material

Supplementary Figure 1 UMAP clustering of *Aire* wild-type TECs based on their transcriptional profile of mRNAs markers *Epcam*, *Itgb4*, *Itga6*, and *Casp3*.

Supplementary Figure 2 UMAP clustering of *Aire* wild-type proliferative TECs based on their transcriptional profile of mRNAs markers *Ccna2*, *Pbk*, and *Birc5*.

Supplementary Figure 3 Heatmap of the expression profiles of the top mRNAs characterizing *Aire* wild-type mTECs, TEC1, TECII, and wild-type naïve CD4^+^ thymocytes.

Supplementary Figure 4 Volcano plot of the 2,000 variable and 17,837 nonvariable mRNAs among *Aire* wild-type TECs, mTECs, and wild-type naïve CD4^+^ thymocytes.

Supplementary Figure 5 Functional annotation of the differentially expressed mRNAs among clusters of *Aire* wild-type TECs, mTECs, and wild-type naïve CD4^+^ thymocytes.

Supplementary Figure 6 Heatmaps of the expression profiles of cell adhesion and transcriptional regulator mRNAs among cell clusters of *Aire* wild-type TECs, mTECs, and wild-type naïve CD4^+^ thymocytes. The mRNAs highlighted in gray represent the mRNAs detected in the interaction network of *Aire* WT mTECs (see **Figure 4**).

Supplementary Figure 7 Identification and percentage expression **(A)** and heatmap **(B)** of individual lncRNAs that are expressed by specific cell types of *Aire* wild-type TECs, mTECs, and wild-type naïve CD4^+^ thymocytes.

Supplementary Figure 8 AIRE protein expression in mTECs. Western-blot of the AIRE protein comparing the WT mTEC 3.10 (WT) with *Aire*-deficient (CS8D6) mTECs. The *Aire-*deficient CS8D6 clone expressed lower amounts of the ~57 kDa AIRE protein. The remaining amount of AIRE observed in the *Aire*-deficient cells may be due to the expression of the *Aire* WT allele.

Supplementary Figure 9 UMAP clustering of *Aire* deficient TECs based on their transcriptional profiles of mRNA markers *Epcam, Itgb4, Itga6, and Casp3*.

Supplementary Figure 10 UMAP clustering of proliferative *Aire* deficient TECs based on their transcriptional profiles of mRNA markers *Ccna2, Pkb, and Birc5*.

Supplementary Figure 11 UMAP clustering of naïve CD4^+^ thymocytes based on their transcriptional profiles of mRNA markers *Ccr7 and Il7r*.

Supplementary Figure 12 Heatmap of the expression profile of *Aire* deficient TECs, TEC I, TEC II, and wild-type naïve CD4^+^ thymocytes.

Supplementary Figure 13 Volcano plot of the 2,000 variable and 17,145 nonvariable mRNAs among *Aire* deficient TECs, TEC I, TEC II, and wild-type naïve CD4^+^ thymocytes.

Supplementary Figure 14 Functional annotation of the differentially expressed mRNAs among cell clusters of TECs, TEC I, TEC II, and wild-type naïve CD4^+^ thymocytes.

Supplementary Figure 15 Heatmaps of the expression profiles of cell adhesion and transcriptional regulators among cell clusters of Aire deficient TECs, TEC I, TEC II, and wild-type naïve CD4^+^ thymocytes. The mRNAs highlighted in gray represent the mRNAs detected in the interaction network of Aire-deficient mTECs (see **Figure 5**).
