# Supplementary figures and images for: The single-cell transcriptome of mTECs and CD4+ thymocytes under adhesion revealed heterogeneity of mTECs and a network controlled by Aire and lncRNAs
Source: Front Immunol. 2024 Aug 26;15:1376655. doi: 10.3389/fimmu.2024.1376655 (PMC11425717; doi:10.3389/fimmu.2024.1376655)

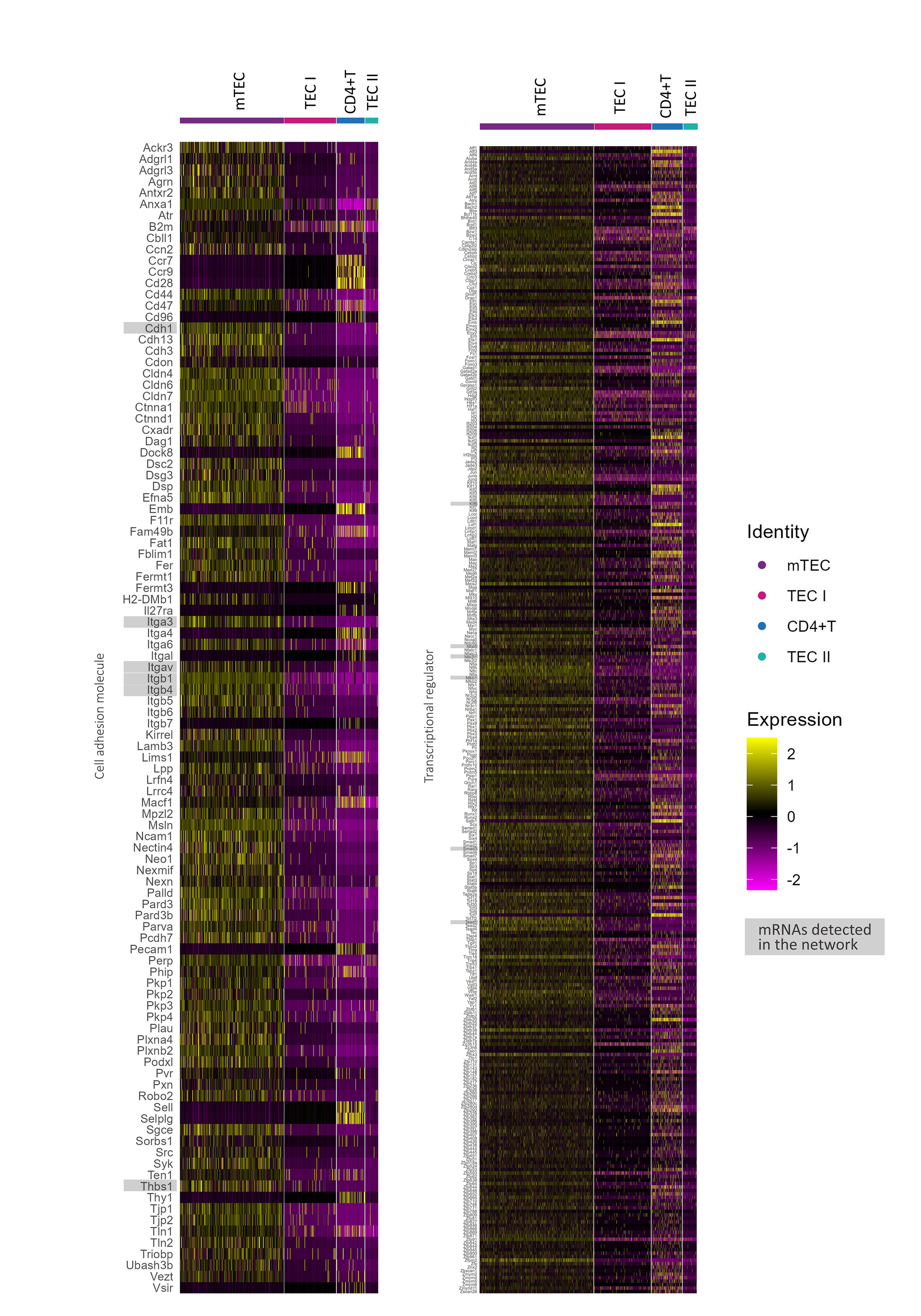

Supplement: Supplementary Figure 6 — Heatmaps of the expression profiles of cell adhesion and transcriptional regulator mRNAs among cell clusters of Aire wild-type TECs, mTECs, and wild-type naïve CD4+ thymocytes. The mRNAs highlighted in gray represent the mRNAs detected in the interaction network of Aire WT mTECs (see Figure 4 ). [file Image6.jpg]

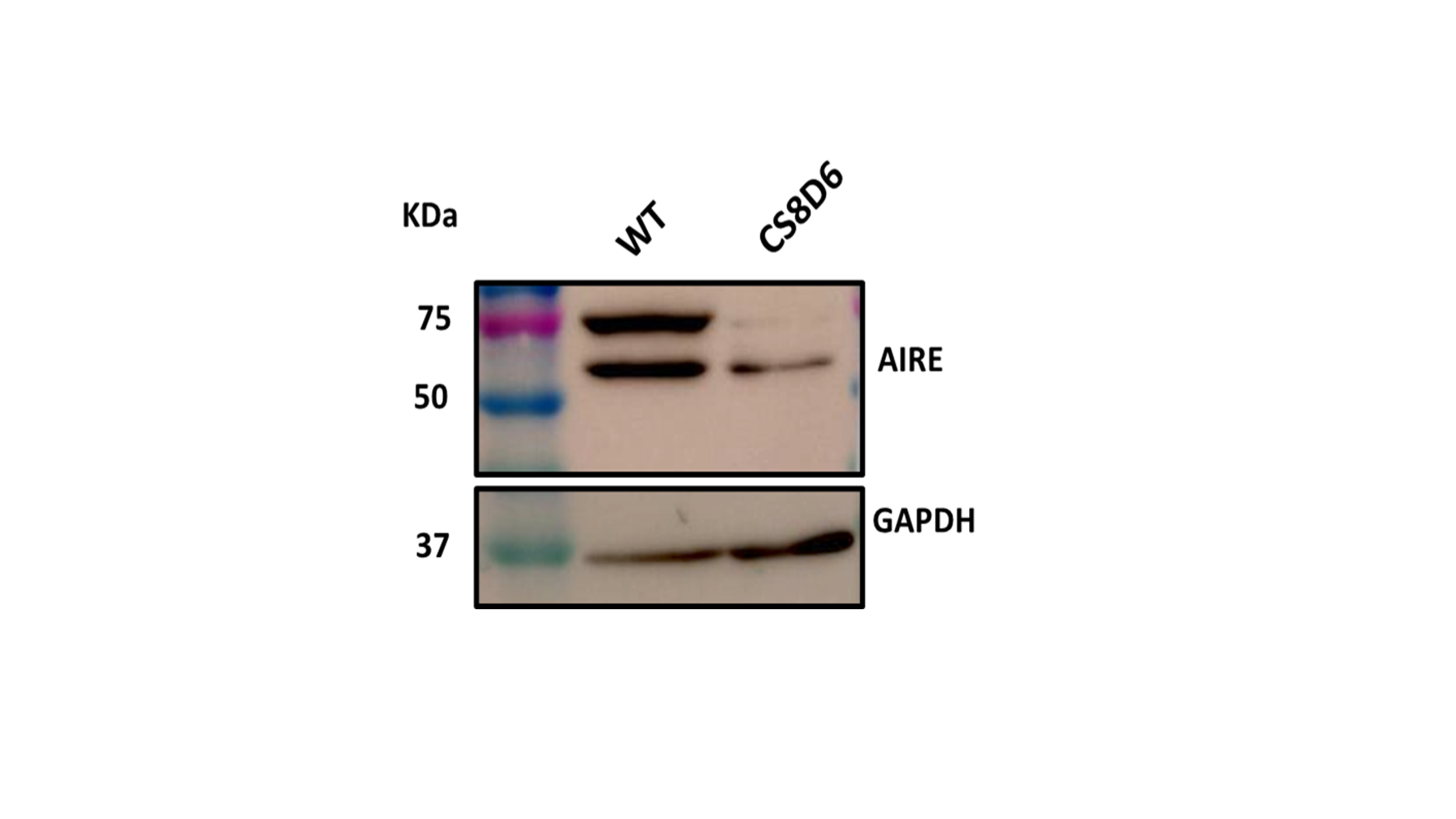

Supplement: Supplementary Figure 8 — AIRE protein expression in mTECs. Western-blot of the AIRE protein comparing the WT mTEC 3.10 (WT) with Aire-deficient (CS8D6) mTECs. The Aire-deficient CS8D6 clone expressed lower amounts of the ~57 kDa AIRE protein. The remaining amount of AIRE observed in the Aire-deficient cells may be due to the expression of the Aire WT allele. [file Image8.tif]

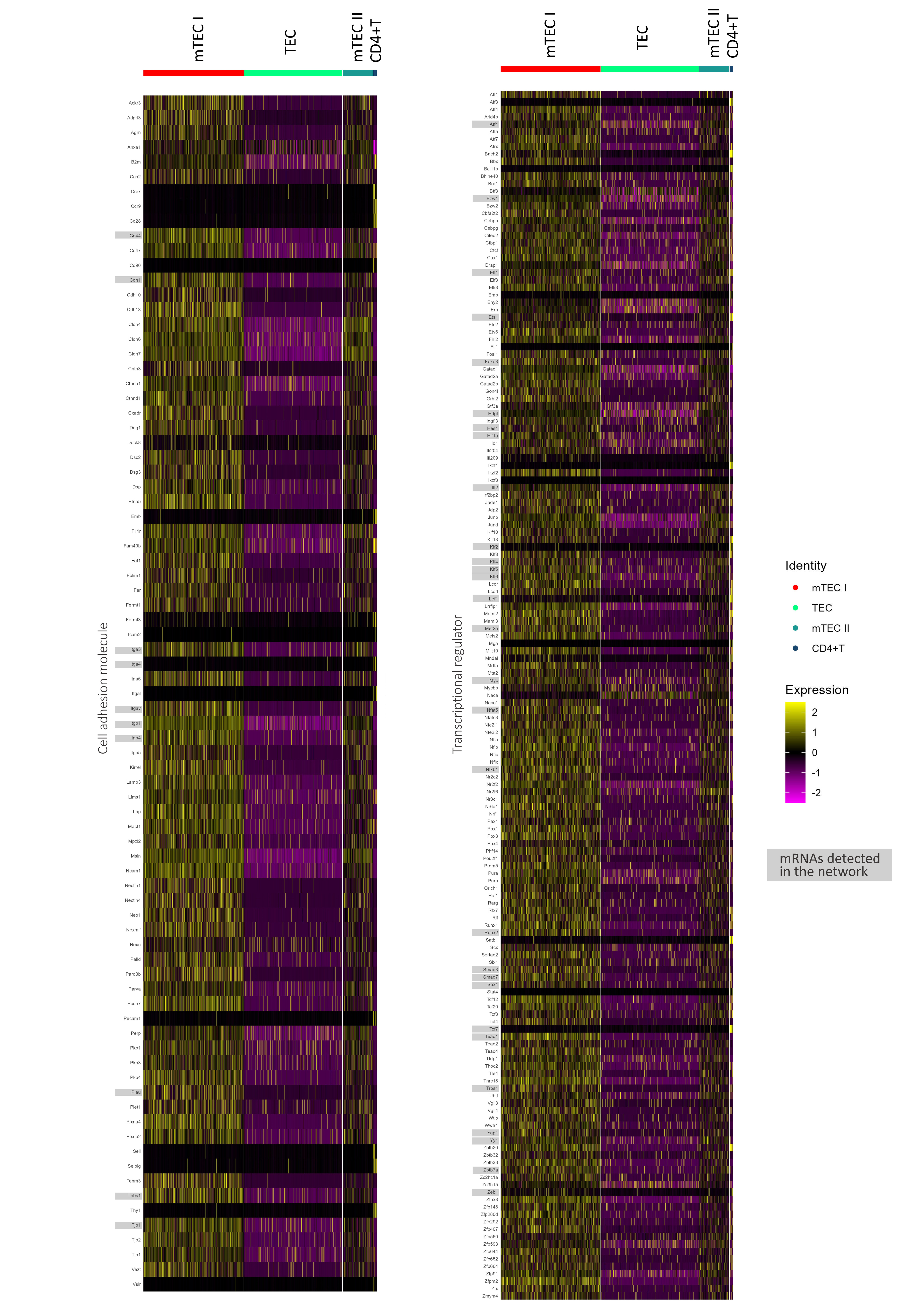

Supplement: Supplementary Figure 15 — Heatmaps of the expression profiles of cell adhesion and transcriptional regulators among cell clusters of Aire deficient TECs, TEC I, TEC II, and wild-type naïve CD4+ thymocytes. The mRNAs highlighted in gray represent the mRNAs detected in the interaction network of Aire-deficient mTECs (see Figure 5 ). [file Image15.jpg]
